# Sixteen new species of the genus *Pseudopoda* Jäger, 2000 from China, Myanmar, and Thailand (Sparassidae, Heteropodinae)

**DOI:** 10.3897/zookeys.791.28137

**Published:** 2018-10-22

**Authors:** Tongyao Jiang, Qingyuan Zhao, Shuqiang Li

**Affiliations:** 1 Institute of Zoology, Chinese Academy of Science, Beijing 100101, China Institute of Zoology, Chinese Academy of Science Beijing China

**Keywords:** Description, huntsman spiders, taxonomy

## Abstract

Sixteen new species of *Pseudopoda* Jäger, 2000 (Sparassidae, Heteropodinae) are described. Among them, eight species were collected from China: *P.chayuensis* Zhao & Li, **sp. n.** (♂), *P.conaensis* Zhao & Li, **sp. n.** (♂), *P.medogensis* Zhao & Li, **sp. n.** (♂), *P.nyingchiensis* Zhao & Li, **sp. n.** (♂), *P.shacunensis* Zhao & Li, **sp. n.** (♂), *P.shuo* Zhao & Li, **sp. n.** (♂♀), *P.yuanjiangensis* Zhao & Li, **sp. n.** (♀) and *P.zixiensis* Zhao & Li, **sp. n.** (♂); seven from Myanmar: *P.colubrina* Zhao & Li, **sp. n.** (♂♀), *P.daxing* Zhao & Li, **sp. n.** (♂), *P.gexiao* Zhao & Li, **sp. n.** (♂), *P.putaoensis* Zhao & Li, **sp. n**. (♂), *P.subbirmanica* Zhao & Li, **sp. n.** (♂♀), *P.titan* Zhao & Li, **sp. n.** (♂♀), *P.xia* Zhao & Li, **sp. n.** (♂); and one from Thailand: *P.maeklongensis* Zhao & Li, **sp. n.** (♂). A distribution map of the new species is also provided.

## Introduction

*Pseudopoda* Jäger, 2000 is currently the third largest genus in the family Sparassidae Bertkau, 1872, containing 124 known species. A molecular phylogeny of Sparassidae asserted that *Pseudopoda* belongs to the subfamily Heteropodinae, and is closely related to *Heteropoda* Latreille, 1804 and *Sinopoda* Jäger, 1999 (Moradmand et al. 2014). Along with the description of 49 new species from Himalayas and adjacent mountains, Jäger (2001) proposed six species-groups mainly according to the features of male pedipalp and female epigyne: *P.diversipunctata*-group, *P.latembola*-group, *P.martensi*-group, *P.parvipunctata*-group, *P.prompta*-group and *P.schwendingeri*-group. Based on both molecular and morphological characteristics, Zhang et al. (2017) proposed the seventh species group: *P.daliensis*-group and described three new species from Yunnan, China.

Currently, all of the *Pseudopoda* species are found in Asian countries: Bhutan, China, India, Indonesia, Japan, Laos, Myanmar, Nepal, Pakistan, Thailand, and Vietnam. To date, 54 species have been reported from China, while 14 from Myanmar and six species from Thailand (World Spider Catalog 2018). A considerable number of them are recorded from high altitude mountain regions, such as the Himalayas and Yunnan-Guizhou Plateau in China. Most of the *Pseudopoda* species exhibit very small-ranged distributions, but a high local diversity. A previous research explored on the application of DNA barcoding in taxonomic assessment in this genus, and indicated there is a greater species diversity remaining to be discovered (Cao et al. 2016). Here, we described 16 newly discovered species collected from southern China (Yunnan Province, Jiangxi Province and Tibet Autonomous Region), northern Myanmar (Kachin State), and Thailand (Tak Province).

## Material and methods

All specimens were examined and measured with a Leica M205C stereomicroscope. Images of male pedipalps and female epigynes were taken with an Olympus C7070 wide zoom digital camera (7.1 megapixels) mounted on an Olympus BX51 compound light microscope after removing them from the spiders’ bodies. Images of bodies were taken with an Olympus C7070 camera mounted on an Olympus SZX12 dissecting microscope. Epigynes were cleaned and treated in trypsin and if necessary, in boiling solution of potassium hydroxide (KOH) before taking images of the vulvae. All images were assembled using Helicon Focus 6.7.1 software.

All measurements are in millimeters. Leg formula, spination, and measurements of palp and legs follow Jäger and Vedel (2007). Arising points of tegular appendices (i.e. embolus, conductor) are given as ‘clock positions’ on the left palp in ventral view. When the left palp is lost or incomplete, the images of right palp will be taken and flipped horizontally for the sake of comparison. In this case, the right palp will be treated as the left one.

Abbreviations used in the text and figures are given below:

All material studied are deposited in the Institute of Zoology, Chinese Academy of Sciences (**IZCAS**) in Beijing, China.

## Taxonomy

### Family Sparassidae Bertkau, 1872

#### Subfamily Heteropodinae Thorell, 1873

##### Genus Pseudopoda

Taxon classificationAnimaliaAraneaeSparassidae

Jäger, 2000

###### Type Species.

*Sarotespromptus* O. Pickard-Cambridge, 1885

###### Diagnosis.

Exclusively distributed in Asia. Small to large Heteropodinae. Male palp with membranous conductor (but sometimes absent), embolus at least in its basal part broadened and flattened, RTA arising basally or mesially from tibia; female epigyne with lateral lobes rising distantly beyond epigastric furrow, and in most cases covering median septum (modified from Jäger 2000).

##### Pseudopoda chayuensis

Taxon classificationAnimaliaAraneaeSparassidae

Zhao & Li sp. n.

http://zoobank.org/16E0E430-38B3-4913-A1C0-08A3ACA430FB

Figs 1 , 2 , 37

###### Type material.

**Holotype** ♂: China, Tibet Autonomous Region, Nyingchi Prefecture, Chayu County, Walong, 28°35.092'N, 98°07.384'E, 3680 m, VIII 2013, J. Liu.

###### Etymology.

The specific name refers to the type locality; adjective.

###### Diagnosis.

**Figure 1.**
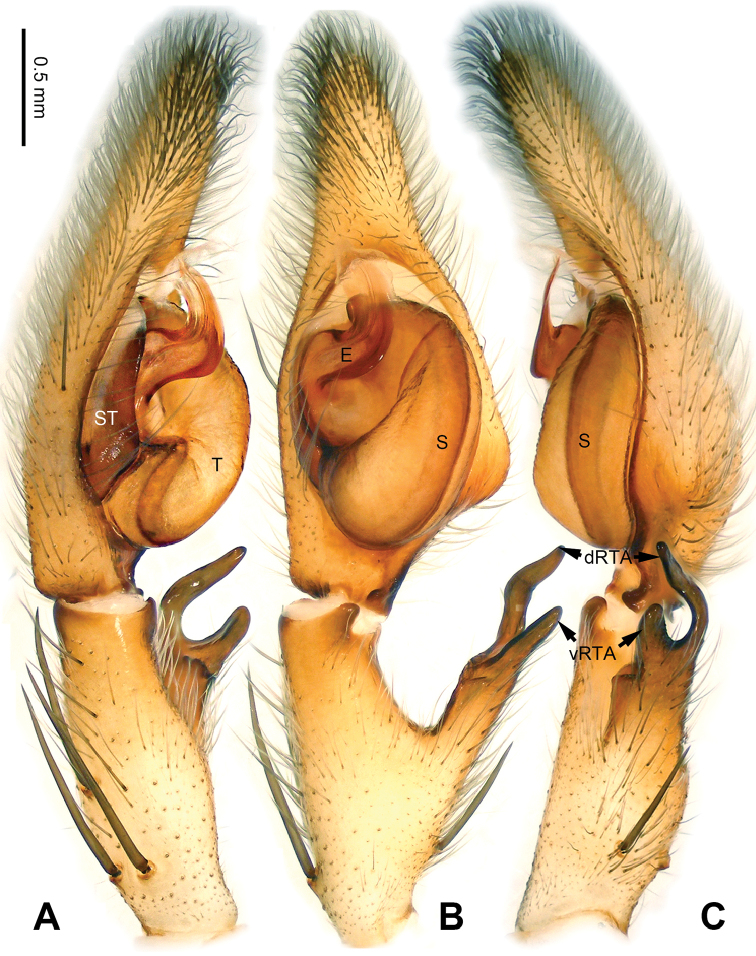
*Pseudopodachayuensis* Zhao & Li, sp. n., right palp of male holotype, horizontally flipped for the sake of comparison. **A** Prolateral view **B** Ventral view **C** Retrolateral view. Scale bar equal for **A, B, C**.

**Figure 2.**
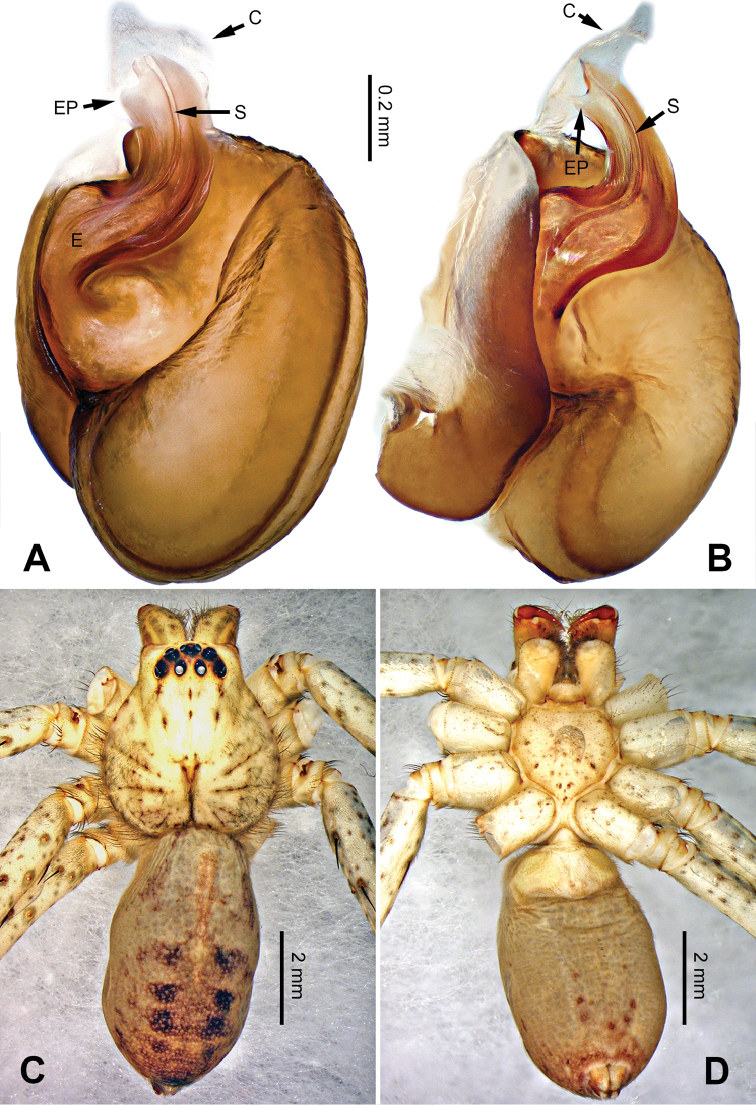
*Pseudopodachayuensis* Zhao & Li, sp. n., male holotype. Right bulb horizontally flipped for the sake of comparison. **A** Bulb, ventral view **B** Bulb, dorsal view **C** Habitus, dorsal view **D** Habitus, ventral view. Scale bar equal for **A, B**.

Medium-sized *Pseudopoda* species. Male resembles *P.gongschana* Jäger & Vedel, 2007 (see Jäger and Vedel 2007: 6, figs 10–15) and *P.platembola* Jäger, 2001 (see Jäger 2001: 57, figure 35a–e) by: 1. embolus sickle-shaped, tapering very moderately (Figure 2A); 2. dRTA well developed and finger-like, curving distally (Figure 1A–C). It can be distinguished from the two congeners by the following combination of characters: 1. embolic projection near the tip of embolus, making the tip look somewhat incised (Figure 1A; embolic projection absent in *P.platembola*); 2. embolus curving more intensely than in *P.gongschana* (Figure 2A).

###### Description.

Male (holotype). Body length 10.7, DS length 4.3, DS width 4.1, OS length 6.4, OS width 3.4. Eyes: AME 0.16, ALE 0.24, PME 0.16, PLE 0.30, AME-AME 0.20, AME-ALE 0.10, PME-PME 0.33, PME-PLE 0.33, AME-PME 0.41, ALE-PLE 0.33, CH AME 0.32, CH ALE 0.24. Leg formula: II-I-IV-III. Spination: palp 131, 101, 2111; legs: femur I-III 323, IV 331; patella I-IV 001; tibia I-IV 2126; metatarsus I-II 2024, III 3025, IV 3037. Measurements of palp and legs: palp - (-, 1.0, 1.4, -, 2.4), leg I 26.3 (7.0, 2.5, 7.0, 7.5, 2.3), leg II 28.3 (7.5, 2.5, 8.0, 8.0, 2.3), leg III 23.2 (6.8, 2.3, 6.1, 6.1, 1.9), leg IV 25.7 (7.0, 2.1, 6.8, 7.5, 2.3). Promargin of chelicerae with three teeth, retromargin with four teeth. Cheliceral furrow with ca. 15 denticles.

Palp as in diagnosis. Cymbium distally slender and elongated, with a small retrobasal projection in ventral view. RTA arising basally to mesially from tibia, vRTA thumb-like, shorter than dRTA (Figure 1A–C). Sperm duct running submarginally retrolaterally in tegulum. Embolus sickle-shaped, arising from tegulum at 10 o’clock position. The embolus tapering and very moderately curved. Embolic projection emerging at the prolateral margin of embolus as a blunt hump. Conductor arising from tegulum at 12 o’clock position, slightly leaning prolaterally and covering the tip of embolus (Figure 2A, B).

Coloration in ethanol: carapace yellow. Radial furrows and fovea dark brown. Dorsal opisthosoma brown with black pattern. Legs yellowish brown, with reddish brown dots and patches (Figure 2C, D).

Female. Unknown.

###### Distribution.

Known only from the type locality.

##### Pseudopoda colubrina

Taxon classificationAnimaliaAraneaeSparassidae

Zhao & Li sp. n.

http://zoobank.org/8EDAFE92-8991-4BD0-A68F-C3895F419AB5

Figs 3 , 4 , 5 , 37

###### Type material.

**Holotype** ♂: Myanmar, Kachin State, Putao, road to Ziradum Village, 27°33.617'N, 97°06.567'E, 1003 m, 8 V 2017, J. Wu & Z. Chen. **Paratype**: 1 ♀, same locality as holotype, 13 XII 2016, J. Wu.

###### Etymology.

The specific name is derived from the Latin word *colubrinus, -a, -um*, meaning ‘serpentine, winding’, and referring to the shape of embolus in this species, which coils at the basal part and erects distally and looks like an alarmed snake; adjective.

###### Diagnosis.

**Figure 3.**
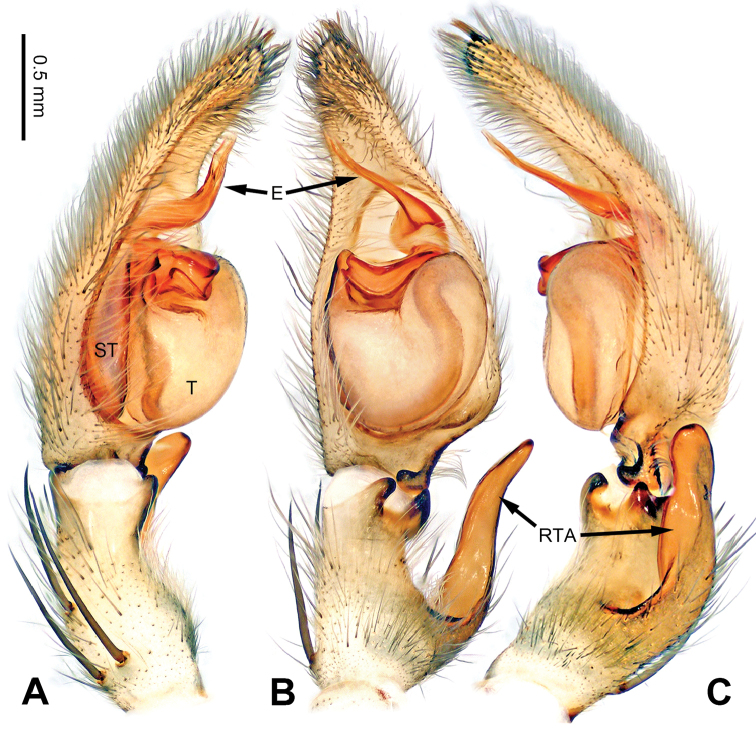
*Pseudopodacolubrina* Zhao & Li, sp. n., left palp of male holotype. **A** Prolateral view **B** Ventral view **C** Retrolateral view. Scale bar equal for **A, B, C**.

**Figure 4.**
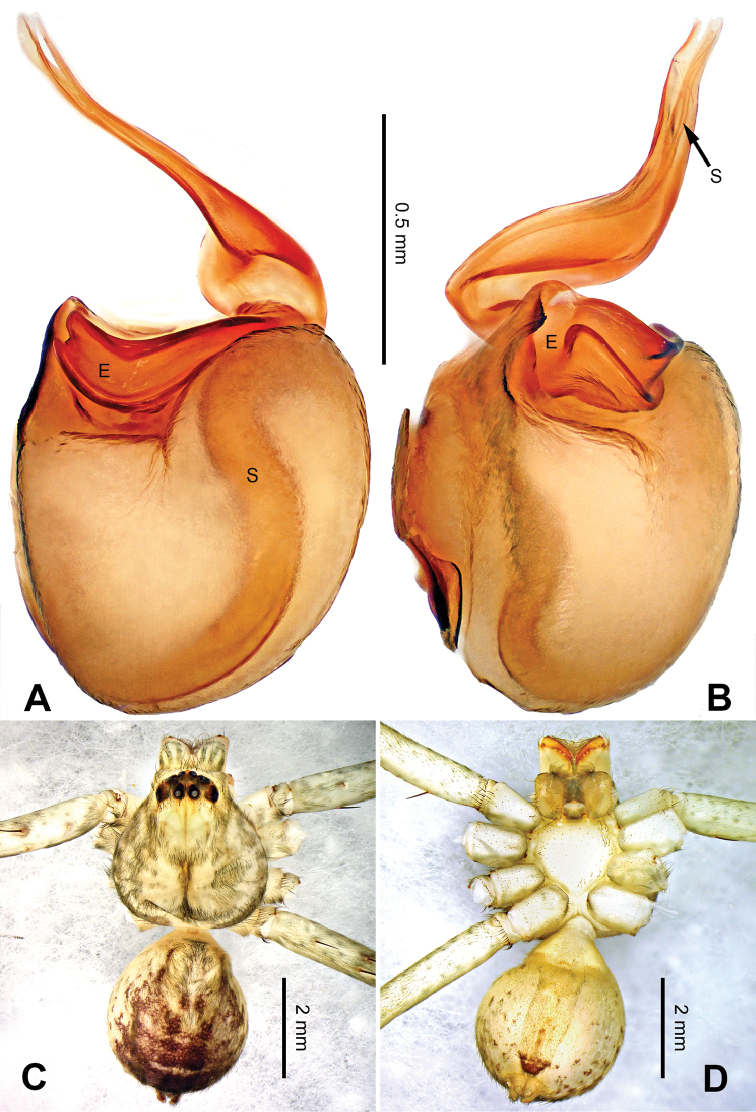
*Pseudopodacolubrina* Zhao & Li, sp. n., male holotype. **A** Bulb, ventral view **B** Bulb, dorsal view **C** Habitus, dorsal view **D** Habitus, ventral view. Scale bar equal for **A, B.**

Small to median-sized *Pseudopoda* species. Male resembles *P.wu* Jäger, Li & Krehenwinkel, 2015 (see Jäger et al. 2015: 384, figs 115–129) and *P.tji* Jäger, 2015 (see Jäger 2015: 333, figs 1–15, 91) by: 1. embolus robust but twisted, forming loops (Figure 4A, B; rarely seem in other *Pseudopoda* spp.); 2. conductor absent (Figure 4A, B). It can be easily distinguished from the two congeners by the following combination of characters: 1. only basal part of embolus twisted, distal part elongated and mildly bent (Figure 4A, B; distal part coiled in *P.tji* and *P.wu*); 2. tegulum occupying two third of alveolus (Figure 3B; covering whole or most of alveolus in *P.tji* and *P.wu*).

**Figure 5.**
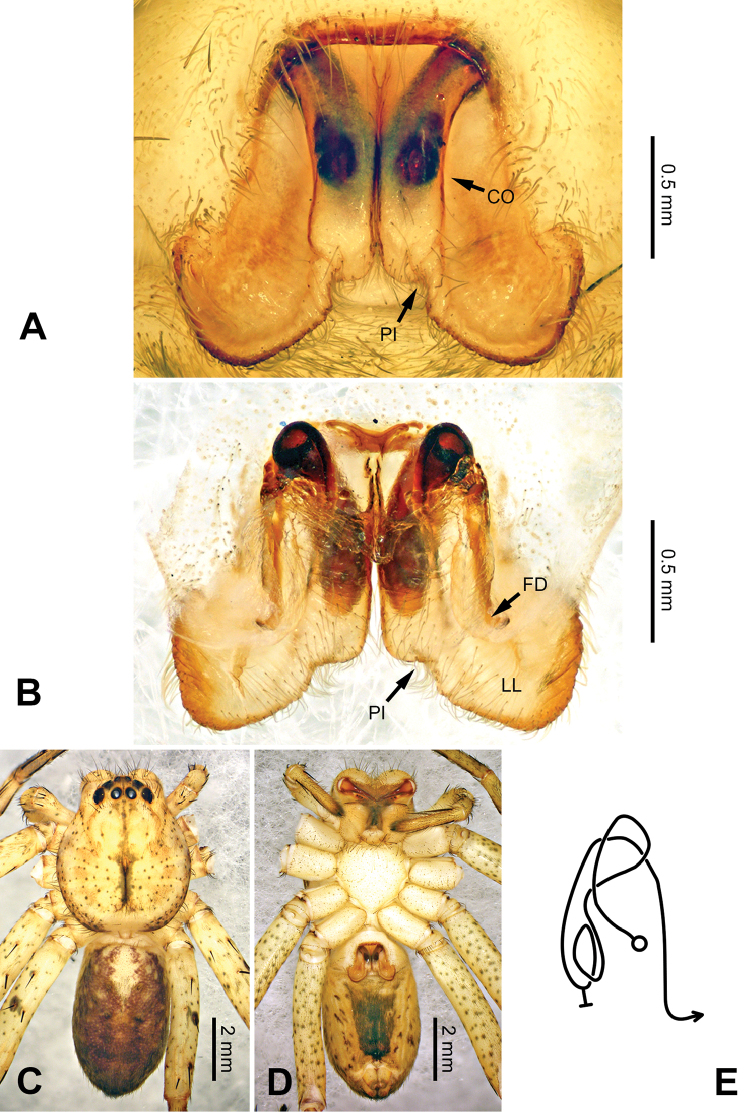
*Pseudopodacolubrina* Zhao & Li, sp. n., paratype female. **A** Epigyne, ventral view **B** Vulva, dorsal view **C** Habitus, dorsal view **D** Habitus, ventral view **E** Schematic course of internal duct system, dorsal view.

Female resembles *P.hyatti* Jäger, 2001 (see Jäger 2001: 72, figs 41j–m, 84) by: 1. posterior part of lateral lobes surpassing the epigastric furrow; 2. loops of internal duct system mainly winding near the central axis, running transversally (Figure 5A, B, E). It can be distinguished from the latter by the following combination of characters: 1. copulatory opening located at the middle to posterior part of epigyne (Figure 5A; located near the anterior margin of lateral lobe in *P.hyatti*); 2. anterior margin of epigynal field truncated, anterior bands absent (Figure 5A; anterior margin of epigynal field trilobate with short anterior bands in *P.hyatti*).

###### Description.

Male (holotype). Body length 8.8, DS length 4.3, DS width 4.1, OS length 4.5, OS width 4.0. Eyes: AME 0.17, ALE 0.34, PME 0.29, PLE 0.28, AME-AME 0.23, AME-ALE 0.09, PME-PME 0.16, PME-PLE 0.33, AME-PME 0.37, ALE-PLE 0.32, CH AME 0.60, CH ALE 0.39. Leg formula: II-I-IV-III. Spination: palp 131, 101, 2101; legs: femur I-II 323, III 322, IV 321; patella I-IV 101; tibia I-II 2026, III 2126, IV 2026; metatarsus I-II 1014, III 2025, IV 3036. Measurements of palp and legs: palp 6.3 (2.0, 0.9, 1.1, -, 2.3), leg I 20.4 (5.8, 2.0, 5.9, 4.9, 1.8), leg II 22.1 (6.1, 2.3, 6.4, 5.4, 1.9), leg III 16.7 (5.0, 1.8, 4.5, 4.0, 1.4), leg IV 19.6 (5.6, 1.7, 5.0, 5.5, 1.8). Promargin of chelicerae with three teeth, retromargin with four teeth. Cheliceral furrow with ca. 21 denticles.

Palp as in diagnosis. Cymbium slender, tip slightly bent prolaterally, with a distinct retrobasal bulge. RTA arising basally from tibia, simple but elongated, blunt in retrolateral view (Figure 3A–C). Sperm duct bending near the top of tegulum, then running submarginally retrolaterally in tegulum. Basal part of embolus with distinct double rims. Tip of embolus pointing distally prolaterally. Conductor completely absent, like a few other species (e.g. *P.ashcharya* Jäger & Kulkarni, 2016) (Figure 4A, B).

Coloration in ethanol: carapace yellowish. Radial furrows and fovea darker brown. Dorsal opisthosoma reddish brown. Legs yellowish, with randomly distributed brown dots (Figure 4C, D).

Female (paratype). Body length 10.0, DS length 4.9, DS width 4.3, OS length 5.1, OS width 3.2. Eyes: AME 0.22, ALE 0.33, PME 0.25, PLE 0.31, AME-AME 0.20, AME-ALE 0.04, PME-PME 0.20, PME-PLE 0.41, AME-PME 0.40, ALE-PLE 0.37, CH AME 0.51, CH ALE 0.41. Leg formula: II-IV-I-III. Spination: palp 131, 101, 2121, 1014; legs: femur I-II 323, III 322, IV 321; patella I-IV 101; tibia I-IV 2026; metatarsus I 1014, II-III 2024, IV 3036. Measurements of palp and legs: palp 5.3 (1.6, 0.7, 1.0, -, 2.0), leg I 17.6 (4.9, 2.0, 5.0, 4.2, 1.5), leg II 19.2 (5.5, 2.2, 5.5, 4.4, 1.6), leg III 14.9 (4.4, 1.8, 3.9, 3.4, 1.4), leg IV 18.1 (5.5, 1.8, 4.5, 4.6, 1.7). Promargin of chelicerae with three teeth, retromargin with four teeth. Cheliceral furrow with ca. 20 denticles.

Epigyne as in diagnosis. Epigynal field with nearly equal length in transverse and longitudinal axis. Lateral lobes longer in longitudinal axis. Median margin of lateral lobes touching each other medially. Internal duct system with loops looming through the lateral lobes in ventral view (Figure 5A). A pair of small appendages present (Figure 5E).

Coloration in ethanol: As in male, but generally darker with more dots and patches (Figure 5C, D).

###### Distribution.

Known only from the type locality.

##### Pseudopoda conaensis

Taxon classificationAnimaliaAraneaeSparassidae

Zhao & Li sp. n.

http://zoobank.org/532C598C-FB21-4DB2-A3B0-8788DF9343E1

Figs 6 , 7 , 37

###### Type material.

**Holotype** ♂: China, Tibet Autonomous Region, Shannan Prefecture, Cona County, Lewang Bridge to Simuzha Scenic Area, roadside and scenic area, 27°49.571'N, 91°43.756'E, 2793 m, 1 VI 2016, J. Wu.

###### Etymology.

The specific name refers to the type locality; adjective.

###### Diagnosis.

**Figure 6.**
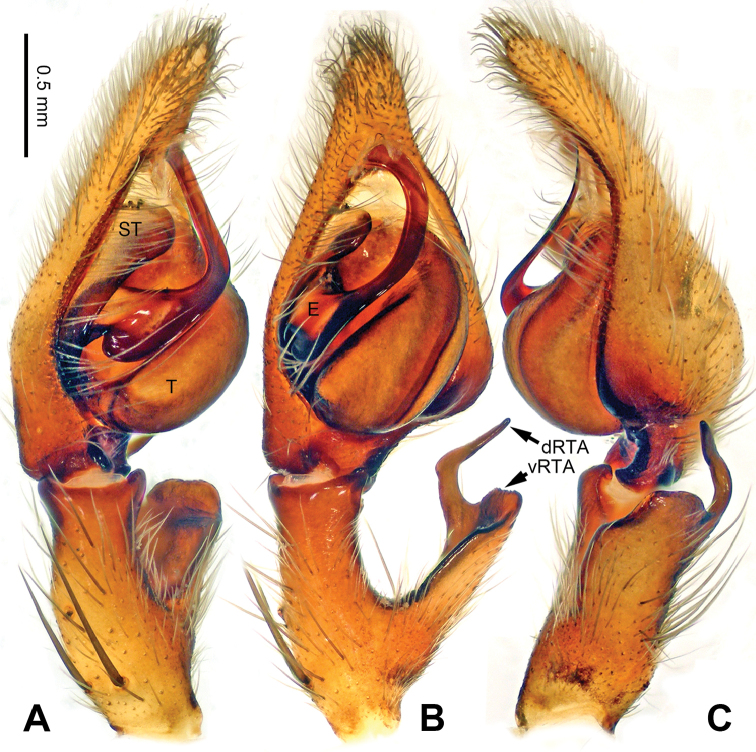
*Pseudopodaconaensis* Zhao & Li, sp. n., left palp of male holotype. **A** Prolateral view **B** Ventral view **C** Retrolateral view. Scale bar equal for **A, B, C**.

**Figure 7.**
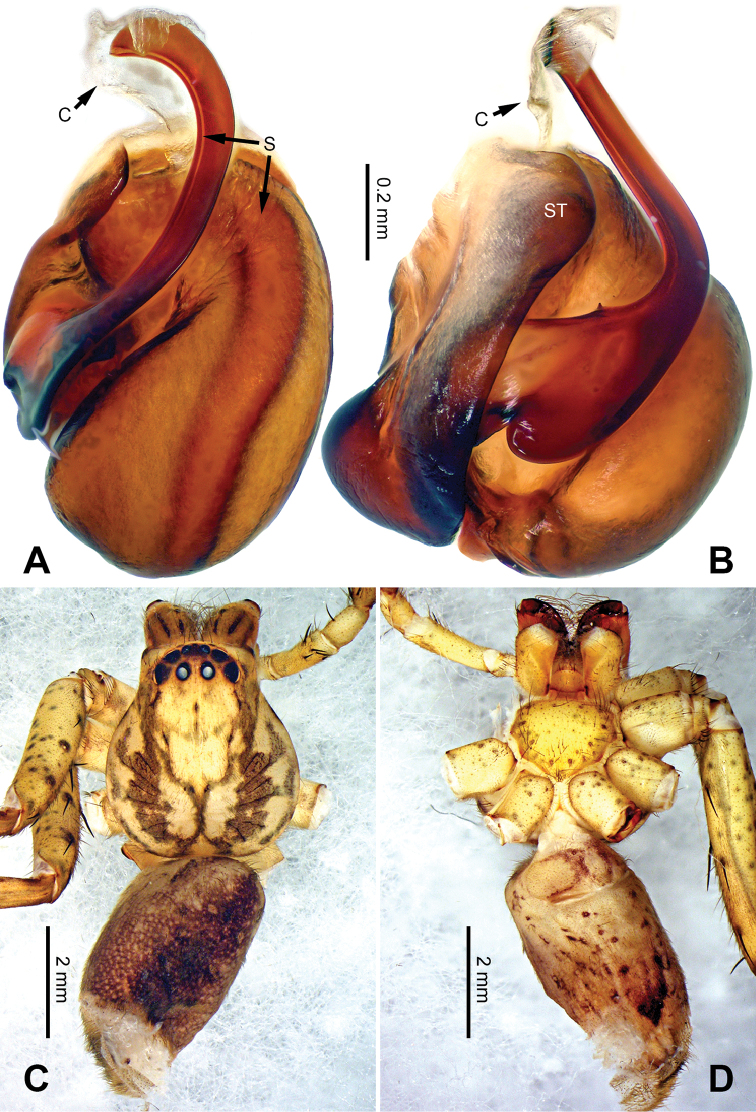
*Pseudopodaconaensis* Zhao & Li, sp. n., male holotype. **A** Bulb, ventral view **B** Bulb, dorsal view **C** Habitus, dorsal view **D** Habitus, ventral view. Scale bar equal for **A, B**.

Small-sized *Pseudopoda* species. Male resembles *P.roganda* Jäger & Vedel, 2007 (see Jäger and Vedel 2007: 18, figs 63–65) and *P.bibulba* (Xu & Yin, 2000) (see Jäger and Vedel 2007: 15, figs 44–59) by: 1. tegulum protruded proximally in retrolateral view; 2. embolus nearly the same width throughout (Figure 7A, B). It can be distinguished from the two congeners by the following combination of characters: 1. basal part of embolus broad (Figure 7B); 2. RTA well developed, dRTA finger-like, bending sharply; vRTA broad, with indention (Figure 6B, C; single-branched RTA in *P.bibulba*; dRTA almost straight in *P.roganda*).

###### Description.

Male (holotype). Body length 8.3, DS length 3.8, DS width 3.1, OS length 4.5, OS width 2.5. Eyes: AME 0.17, ALE 0.25, PME 0.19, PLE 0.26, AME-AME 0.17, AME-ALE 0.06, PME-PME 0.19, PME-PLE 0.30, AME-PME 0.26, ALE-PLE 0.22, CH AME 0.36, CH ALE 0.26. Spination: palp 131, 101, 2101; legs: femur I-II 323, IV 321; patella I-IV 000; tibia I 1026, II-IV 2026; metatarsus I-II 1014, III 3025, IV 3037. Measurements of palp and legs: palp 5.8 (2.0, 0.8, 1.2, -, 1.8), leg I 15.2 (4.0, 1.8, 4.2, 3.8, 1.4), leg II 16.0 (4.3, 1.9, 4.3, 4.0, 1.5), leg III - (-, 1.4, 3.7, 3.6, 1.3), leg IV 15.6 (4.3, 1.6, 3.8, 4.3, 1.6). Promargin of chelicerae with three teeth, retromargin with five teeth. Cheliceral furrow with ca. 22 denticles.

Palp as in diagnosis. Cymbium relatively widened, with distinct retrolateral bulge beside bulb. RTA arising basally from tibia, well developed. Subtegulum extended, covering the base of conductor in prolateral view (Figure 6A–C). Sperm duct running submarginally retrolaterally in tegulum. Embolus long, sickle-shaped, arising from tegulum at 9 o’clock position. Conductor arising from tegulum at 12 o’clock position, leaning prolaterally and covering the tip of embolus (Figure 7A, B).

Coloration in ethanol: carapace yellowish brown, with a pair of dark longitudinal lateral bands. Radial furrows and fovea dark brown. Dorsal opisthosoma reddish brown. Legs yellowish brown, with darker brown dots and patches (Figure 7C, D).

Female. Unknown.

###### Distribution.

Known only from the type locality.

##### Pseudopoda daxing

Taxon classificationAnimaliaAraneaeSparassidae

Zhao & Li sp. n.

http://zoobank.org/993762C9-E4AD-4119-A5D4-4957CD18634A

Figs 8 , 9 , 37

###### Type material.

**Holotype** ♂: Myanmar, Kachin State, Putao, road to Ziradum Village, 27°33.617'N, 97°06.567'E, 1003 m, 13 XII 2016, J. Wu.

###### Etymology.

The specific name is derived from the Chinese Pinyin word for 'large size' (dà xíng), referring to the relatively large body size of the species; noun in apposition.

###### Diagnosis.

Median-sized *Pseudopoda* species. Male resembles those of *P.contraria* Jäger & Vedel, 2007 (Jäger and Vedel 2007: 31, figs 114–119) and *P.semiannulata* Zhang, Zhang & Zhang, 2013 (see Zhang et al. 2013a: 279, figs 13–24) by: 1. embolus extremely expanded, covering nearly half of tegulum; 2. embolus plate-like, with embolic projection on its prolateral margin (Figure 9A, B). It can be distinguished from the two congeners by the following combination of characters: 1. sperm duct running near the prolateral margin of embolus (Figure 9A, B; running near the retrolateral margin in *P.contraria*); 2. tip of embolus and embolic projection slightly bent, pointing distally (Figure 9A; both much more strongly bent in *P.semiannulata*, tip of embolus pointing prolaterally, embolic projection pointing basally).

###### Description.

Male (holotype). Body length 12.4, DS length 6.0, DS width 5.4, OS length 6.4, OS width 3.2. Eyes: AME 0.30, ALE 0.41, PME 0.36, PLE 0.37, AME-AME 0.22, AME-ALE 0.08, PME-PME, 0.26, PME-PLE 0.46, AME-PME 0.44, ALE-PLE 0.43, CH AME 0.57, CH ALE 0.41. Leg formula: II-IV-I-III. Spination: palp 131, 101, 2111; legs: femur I-III 323, IV 321; patella I-IV 001; tibia I-IV 2026; metatarsus I-II 1014, III 2024, IV 3036. Measurements of palp and legs: palp 9.4 (3.1, 1.4, 1.8, -, 3.1), leg I 29.3 (8.3, 3.0, 7.8, 7.8, 2.4), leg II 32.1 (8.7, 3.2, 9.0, 8.5, 2.7), leg III 25.1 (8.0, 2.6, 6.5, 6.0, 2.0), leg IV 29.4 (8.5, 2.5, 7.3, 8.5, 2.6). Promargin of chelicerae with three teeth, retromargin with four teeth. Cheliceral furrow with ca. 25 denticles.

**Figure 8.**
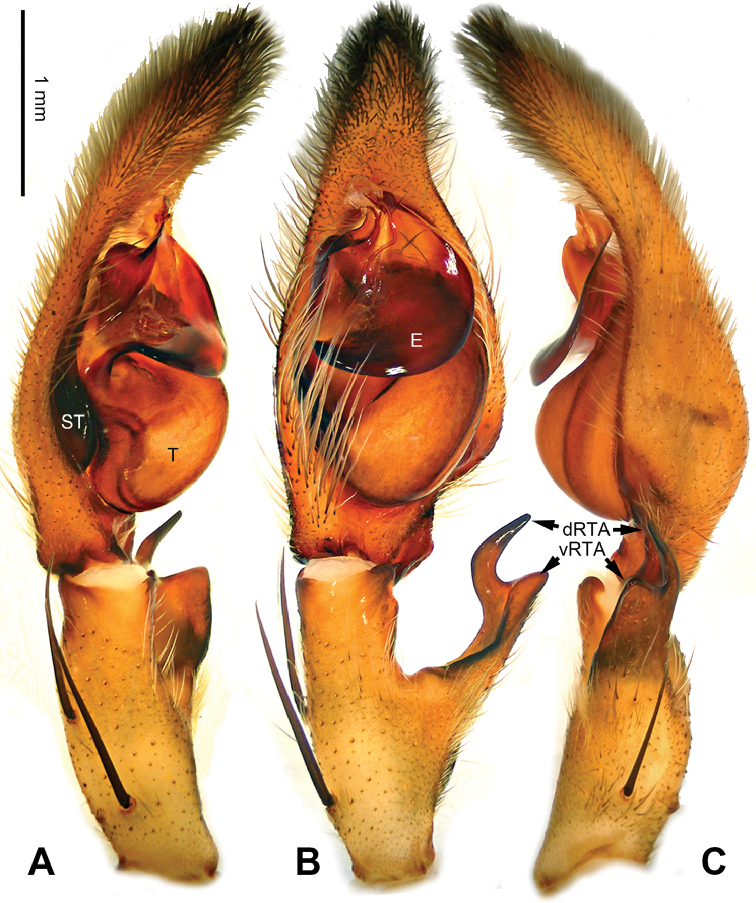
*Pseudopodadaxing* Zhao & Li, sp. n., left palp of male holotype. **A** Prolateral view **B** Ventral view **C** Retrolateral view. Scale bar equal for **A, B, C**.

**Figure 9.**
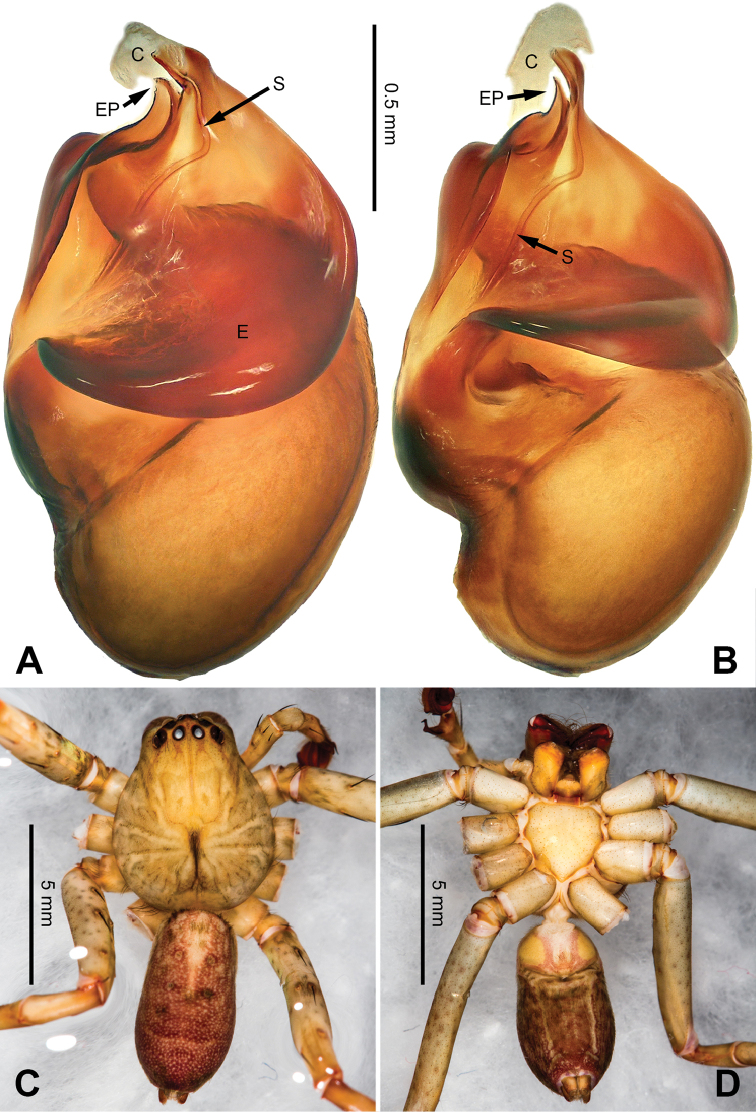
*Pseudopodadaxing* Zhao & Li, sp. n., male holotype. **A** Bulb, ventral view **B** Bulb, dorsal view **C** Habitus, dorsal view **D** Habitus, ventral view. Scale bar equal for **A, B**.

Palp as in diagnosis. Cymbium slender, with retrolateral bulge. RTA arising basally to mesially from tibia, dRTA hook-like, vRTA broad (Figure 8A–C). Sperm duct running submarginally retrolaterally in tegulum, then near the prolateral margin of embolus, meandering like a river flowing around mountains. Embolus arising from tegulum at 9 o’clock position. Conductor arising from tegulum at 12 o’clock position, leaning prolaterally (Figure 9A, B).

Coloration in ethanol: carapace yellowish brown. Radial furrows and fovea dark brown. Dorsal opisthosoma reddish brown. Ventral opisthosoma with a pair of longitudinal bright lines. Legs yellowish brown, with randomly distributed brown dots (Figure 9C, D).

Female. Unknown.

###### Distribution.

Known only from the type locality.

##### Pseudopoda gexiao

Taxon classificationAnimaliaAraneaeSparassidae

Zhao & Li sp. n.

http://zoobank.org/388B9242-F83E-49EE-B8C8-C03F8C1B7336

Figs 10 , 11 , 37

###### Type material.

**Holotype** ♂: Myanmar, Kachin State, Putao, Hponkanrazi Wildlife Sanctuary roadside between Camp 1 to Camp 2, 27°36.067'N, 96°59.367'E, 1714 m, 10 V 2017, J. Wu & Z. Chen. **Paratype**: 1 ♂, same locality as holotype, 17 XII 2016, J. Wu.

###### Etymology.

The specific name is derived from the Chinese Pinyin word for ‘small-size’ (gè xiǎo), referring to the relatively small body size of the species; noun in apposition.

###### Diagnosis.

**Figure 10.**
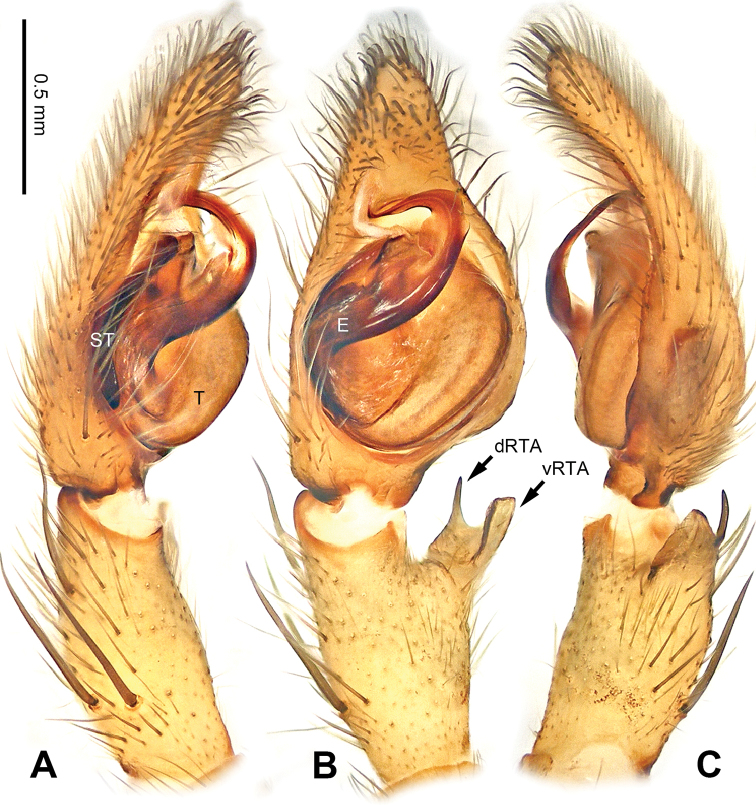
*Pseudopodagexiao* Zhao & Li, sp. n., left palp of male holotype. **A** Prolateral view **B** Ventral view **C** Retrolateral view. Scale bar equal for **A, B, C**.

**Figure 11.**
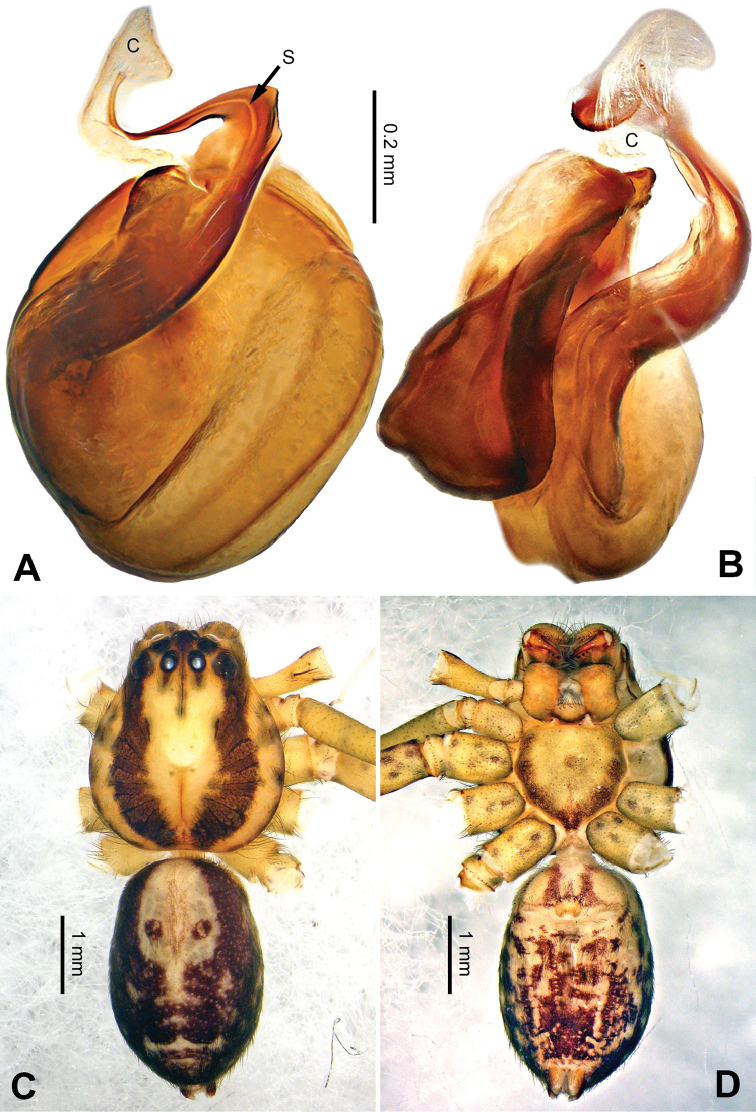
*Pseudopodagexiao* Zhao & Li, sp. n., male holotype. **A** Bulb, ventral view **B** Bulb, dorsal view **C** Habitus, dorsal view **D** Habitus, ventral view. Scale bar equal for **A**, **B**.

Small sized *Pseudopoda* species. Male resembles *P.exigua* (Fox, 1938) (see Jäger 2001: 87, figure 47h–l), *P.grahami* (Fox, 1936) (see Chen and Gao 1990: 156, figure 200a–b) and *P.amelia* Jäger & Vedel, 2007 (see Jäger and Vedel 2007: 12, figs 32–37) by: basal part of embolus broad, while the distal part tapering gradually and becoming filiform at distal end (Figure 11A). It can be distinguished from the three congeners by the following combination of characters: 1. RTA arising mesially from tibia, dividing into dRTA and vRTA (Figure 10B, C; arising basally in *P.grahami*; single-branched RTA in *P.exigua*); 2. tip of embolus bent with its end pointing distally retrolaterally (Figure 11A; bent and pointing prolaterally in *P.amelia*).

###### Description.

Male (measurements of holotype first, those for paratype in parentheses). Body length 5.9 (5.4), DS length 2.8 (3.0), DS width 2.6 (2.6), OS length 3.1 (2.4), OS width 2.0 (1.7). Eyes: AME 0.16 (0.14), ALE 0.26 (0.25), PME 0.15 (0.19), PLE 0.28 (0.25), AME-AME 0.12 (0.13), AME-ALE 0.02 (0.06), PME-PME 0.19 (0.16), PME-PLE 0.21 (0.29), AME-PME 0.26 (0.32), ALE-PLE 0.15 (0.22), CH AME 0.20 (0.21), CH ALE 0.20 (0.15). Spination: palp 131, 101, 2111; legs: femur II-III 323, IV 321; patella I-IV 001; tibia I-III 2026, IV 2126; metatarsus I-II 1014, III 3035, IV 3036. Measurements of palp and legs: palp 3.9 (4.1) (1.1, 0.6, 0.9, -, 1.3), leg I - (-, 1.3, 2.8, 2.5, 1.1), leg II 11.2 (11.4) (3.2, 1.2, 3, 2.6, 1.2), leg III - (10.2) (-, -, -, -, -), leg IV - (11.2) (-, 1.0, 2.7, 3.1, 1.2). Promargin of chelicerae with three teeth, retromargin with four teeth. Cheliceral furrow with ca. 25 denticles.

Palp as in diagnosis. Retrolateral margin of cymbium swollen. Distal part of cymbium sub-triangular. RTA arising mesially to distally from tibia, dRTA needle-like, while vRTA broad (Figure 10A–C). Sperm duct running submarginally retrolaterally in tegulum. Embolus sickle-shaped, arising from tegulum at 9 o’clock position. Basal part of embolus broad, then tapering as it runs and coils, resulting in a filiform tip. Conductor arising from tegulum at 11 o’clock position, leaning prolaterally and then bent in a right angle, with its end covering the tip of embolus (Figure 11A, B).

Coloration in ethanol: carapace yellow, with a pair of dark longitudinal lateral bands. Dorsal opisthosoma reddish brown with a bright transverse band in the posterior half. Legs yellowish brown, with reddish brown dots and patches (Figure 11C, D).

Female. Unknown.

###### Distribution.

Known only from the type locality.

##### Pseudopoda maeklongensis

Taxon classificationAnimaliaAraneaeSparassidae

Zhao & Li sp. n.

http://zoobank.org/5317C261-04E4-443F-A4BB-B2F8EACB0048

Figs 12 , 13 , 37

###### Type material.

**Holotype** ♂: Thailand, Tak Province, Umphang District, Mae Klong Subdistrict, field, 16°14.642'N, 98°59.914'E, 1228 m, 17 XI 2016, H. Zhao, Y. Li & Z. Chen.

###### Etymology.

The specific name refers to the type locality; adjective.

###### Diagnosis.

**Figure 12.**
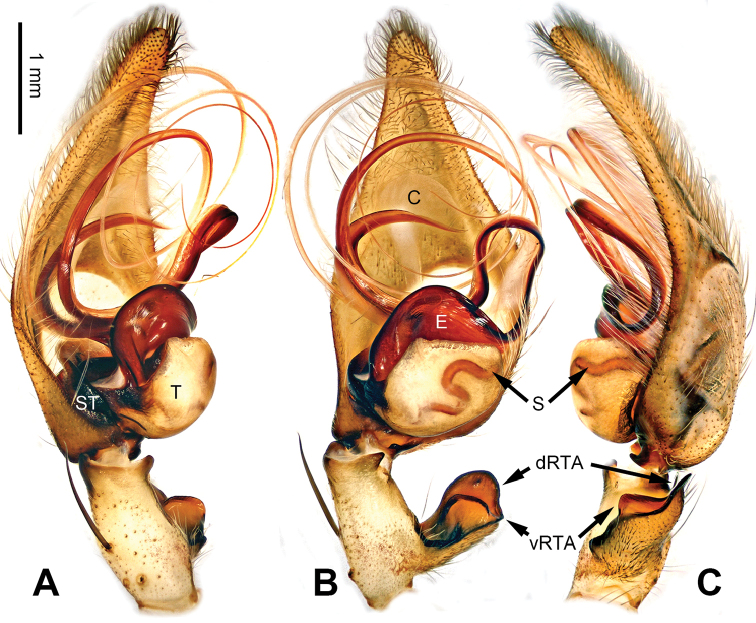
*Pseudopodamaeklongensis* Zhao & Li, sp. n., right palp of male holotype, horizontally flipped for the sake of comparison. **A** Prolateral view **B** Ventral view **C** Retrolateral view. Scale bar equal for **A, B, C**.

**Figure 13.**
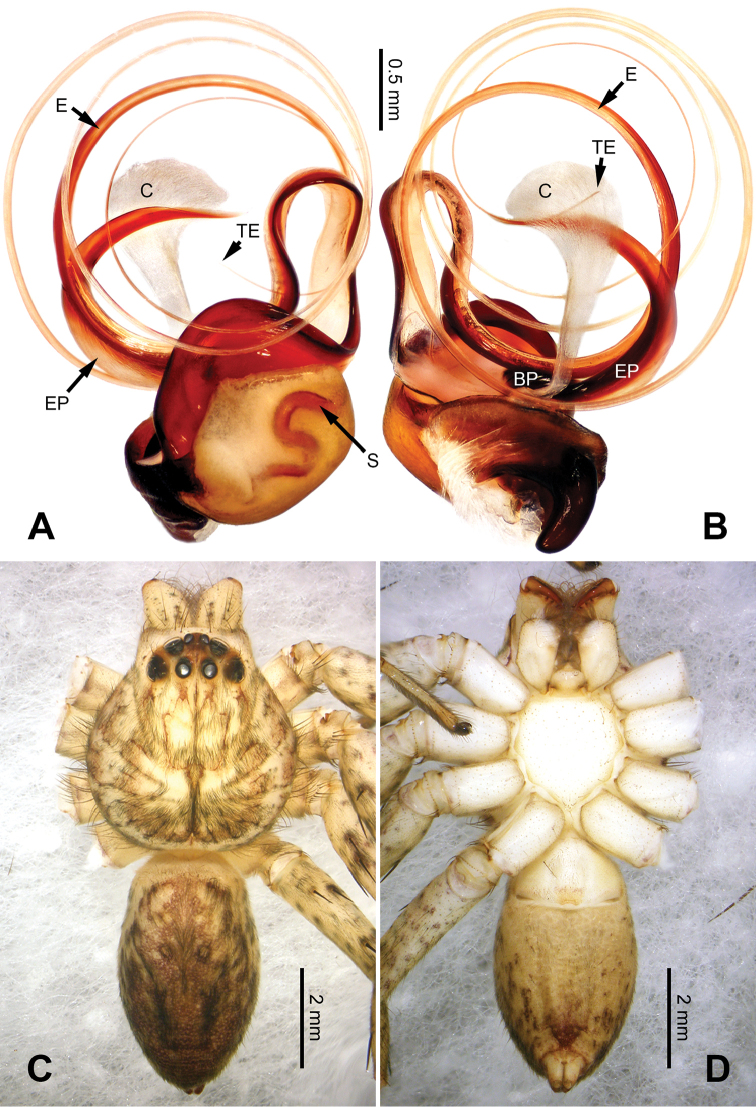
*Pseudopodamaeklongensis* Zhao & Li, sp. n., male holotype. Right bulb horizontally flipped for the sake of comparison. **A** Bulb, ventral view **B** Bulb, dorsal view **C** Habitus, dorsal view **D** Habitus, ventral view. Scale bar equal for **A, B**.

Small-sized *Pseudopoda* species. Male has long spiral embolus that resembles *P.parvipunctata* Jäger, 2001 (see Jäger 2001: 94, figure 49e–l) and *P.spirembolus* Jäger & Ono, 2002 (see Jäger and Ono 2002: 112, figs 11–14). It can be distinguished from the two congeners by the following combination of characters: 1. tegulum small, leaning towards the retrolateral margin of cymbium (Figure 12B); 2. embolic projection long, arising from the basal part of embolus at 9 o’clock position, forming a semicircle with its basal part running along with embolus and covering a part of it like a sheath (Figure 13A, B; absent in *P.spirembolus* and *P.parvipunctata*); 3. embolus extremely long, forming five loops (Figure 13A, B; forming three loops in *P.spirembolus*; two in *P.parvipunctata*); 4. cymbium flattened and broadened without any bulges (Figure 12A–C; elongated and with one bulge on the retrolateral margin in *P.parvipunctata*; broadened and with one bulge on the retrolateral margin in *P.spirembolus*).

###### Description.

Male (holotype). Body length 9.3, DS length 4.4, DS width 4.0, OS length 4.9, OS width 2.8. Eyes: AME 0.21, ALE 0.37, PME 0.26, PLE 0.38, AME-AME 0.16, AME-ALE 0.03, PME-PME 0.22, PME-PLE 0.36, AME-PME 0.43, ALE-PLE 0.32, CH AME 0.45, CH ALE 0.38. Leg formula: II-I-IV-III. Spination: palp 131, 101, 2101; legs: femur I-II 323, III 333, IV 331; patella I-IV 101; tibia I-IV 2026; metatarsus I-II 1014, III 2024, IV 3037. Measurements of palp and legs: palp 8.4 (3.0, 0.8, 1.2, -, 3.4), leg I 21.9 (5.9, 2.4, 6.4, 5.4, 1.8), leg II 23.4 (6.4, 2.5, 6.7, 5.8, 2), leg III 17.2 (5.1, 1.8, 4.8, 4.1, 1.4) leg IV 21.5 (6.2, 1.8, 5.5, 6.2, 1.8). Promargin of chelicerae with three teeth, retromargin with four teeth. Cheliceral furrow with ca. 38 denticles.

Palp as in diagnosis. Cymbium large. RTA arising basally from tibia. Both vRTA and dRTA flattened and blunt in ventral view (Figure 12A–C). Sperm duct S-shaped, running retrolaterally in tegulum. Embolus arising from tegulum at 9 o’clock position, extremely elongated. Conductor large and elongated, arising from the tegulum at 10 to 12 o’clock position (Figure 13A, B).

Coloration in ethanol: carapace yellow. Radial furrows and fovea brown. Dorsal opisthosoma yellowish to reddish brown. Legs yellow, with randomly distributed brown dots (Figure 13C, D).

Female. Unknown.

###### Distribution.

Known only from the type locality.

##### Pseudopoda medogensis

Taxon classificationAnimaliaAraneaeSparassidae

Zhao & Li sp. n.

http://zoobank.org/9C23B103-6026-4856-9CC2-E2874772F9FA

Figs 14 , 15 , 37

###### Type material.

**Holotype** ♂: China, Tibet Autonomous Region, Nyingchi Prefecture, Medog County, 8 km of the road of Beibeng to Gelin, 29°14.660'N, 95°11.442'E, 1235 m, 11 VIII 2017, M. Xu.

###### Etymology.

The specific name refers to the type locality; adjective.

###### Diagnosis.

**Figure 14.**
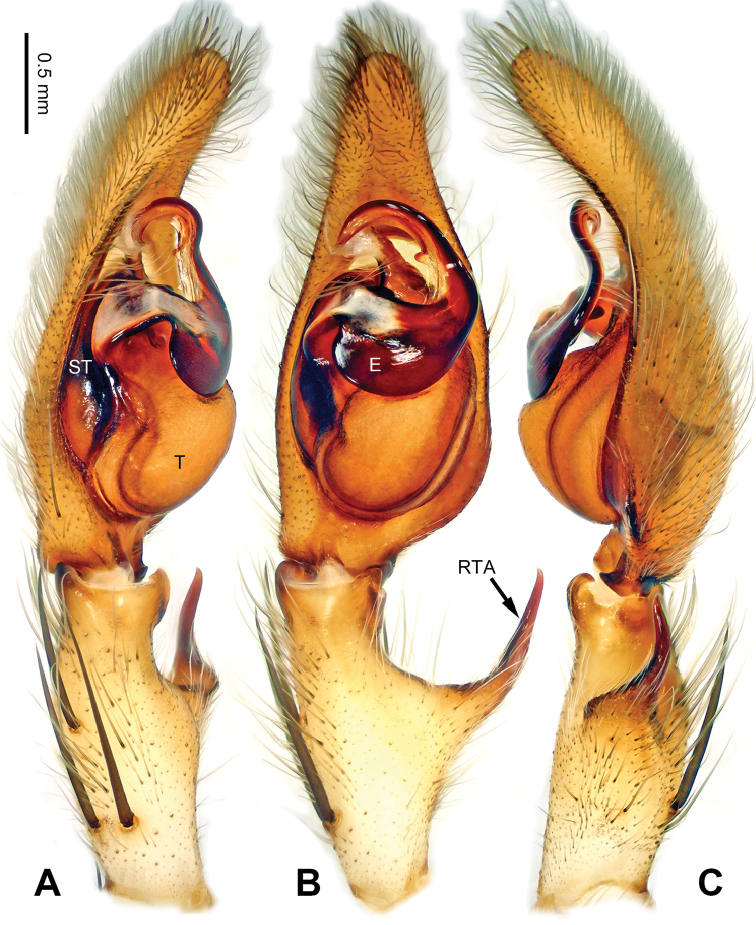
*Pseudopodamedogensis* Zhao & Li, sp. n., left palp of male holotype. **A** Prolateral view **B** Ventral view **C** Retrolateral view. Scale bar equal for **A, B, C**.

**Figure 15.**
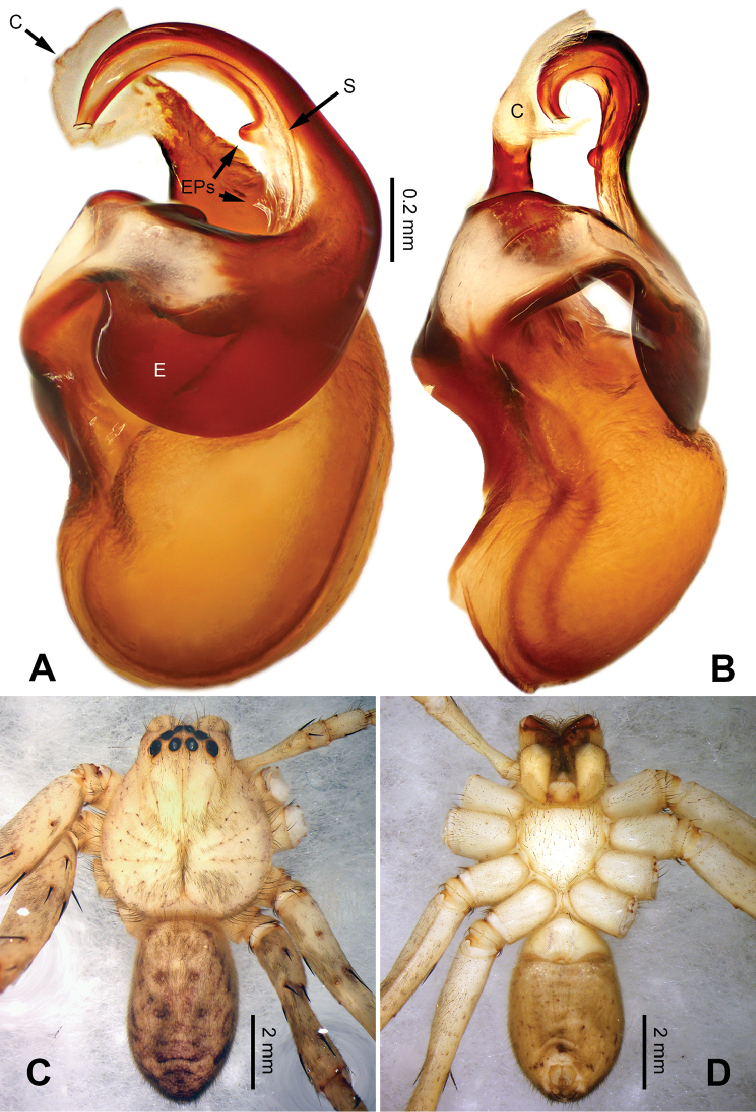
*Pseudopodamedogensis* Zhao & Li, sp. n., male holotype. **A** Bulb, ventral view **B** Bulb, dorsal view **C** Habitus, dorsal view **D** Habitus, ventral view. Scale bar equal for **A, B**.

Median-sized *Pseudopoda* species. Male resembles *P.obtusa* Jäger & Vedel, 2007 (see Jäger and Vedel 2007: 25, figs 91–96) by: embolus broadened at its median part, distal part narrow and curved with embolic projection emerging prolaterally (Figure 15A, B). It can be distinguished from the latter by the following combination of characters: 1. RTA simple and pointed (Figure 14A–C; RTA with humps and blunt apices in *P.obtusa*); 2.distal part of embolus longer, bending more intensely than in *P.obtusa* (Figure 15A, B); 3. two embolic projections on the prolateral margin of distal embolus, the proximal one translucent (Figure 15A; only one on the same margin in *P.obtusa*).

###### Description.

Male (holotype). Body length 10.4, DS length 5.1, DS width 4.7, OS length 5.3, OS width 3.2. Eyes: AME 0.25, ALE 0.40, PME 0.22, PLE 0.35, AME-AME 0.19, AME-ALE 0.06, PME-PME 0.28, PME-PLE 0.40, AME-PME 0.40, ALE-PLE 0.40, CH AME 0.39, CH ALE 0.33. Leg formula: II-I-IV-III. Spination: palp 131, 101, 2100; legs: femur I-III 323, IV 322; patella I-IV 101; tibia I 2126, II 3236, III-IV 2226; metatarsus I-II 1014, III 2025, IV 3037. Measurements of palp and legs: palp 8.6 (3.1, 1.3, 1.6, -, 2.6), leg I 28.2 (7.8, 2.8, 8.0, 7.2, 2.4), leg II 30.8 (8.2, 3.1, 8.8, 8.0, 2.7), leg III 23.9 (6.8, 2.5, 6.7, 6.0, 1.9), leg IV 26.0 (7.3, 2.5, 6.9, 7.0, 2.3). Promargin of chelicerae with three teeth, retromargin with four teeth. Cheliceral furrow with ca. 35 denticles.

Palp as in diagnosis. Cymbium slender. RTA almost straight, arising mesially from tibia (Figure 14A–C). Sperm duct running submarginally retrolaterally in tegulum. Embolus arising from tegulum at 10 to 11 o’clock position with its basal part broadened. Distal part of embolus curved intensely, with its tip pointing at the base of embolus. Conductor arising from tegulum at 11 o’clock position (Figure 15A, B).

Coloration in ethanol: carapace bright brown. Radial furrows and fovea darker. Dorsal opisthosoma dark brown with black pattern. Legs bright brown, with dark brown patches (Figure 15C, D).

Female. Unknown.

###### Distribution.

Known only from the type locality.

##### Pseudopoda nyingchiensis

Taxon classificationAnimaliaAraneaeSparassidae

Zhao & Li sp. n.

http://zoobank.org/42C87FCE-E01A-47E0-9177-30A531AC9673

Figs 16 , 17 , 37

###### Type material.

**Holotype** ♂: China, Tibet Autonomous Region, Nyingchi Prefecture, between Sejila Moution to Bayi Town, 29°33.790'N, 94°34.247'E, 3847 m, 13 VI 2016, J. Wu.

###### Etymology.

The specific name refers to the type locality; adjective.

###### Diagnosis.

**Figure 16.**
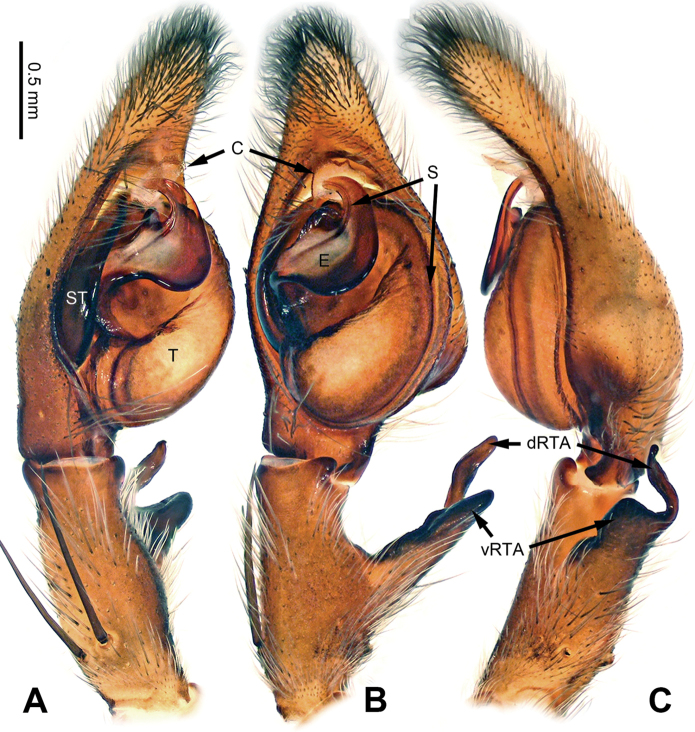
*Pseudopodanyingchiensis* Zhao & Li, sp. n., right palp of male holotype, horizontally flipped for the sake of comparison. **A** Prolateral view **B** Ventral view **C** Retrolateral view. Scale bar equal for **A, B, C**.

**Figure 17.**
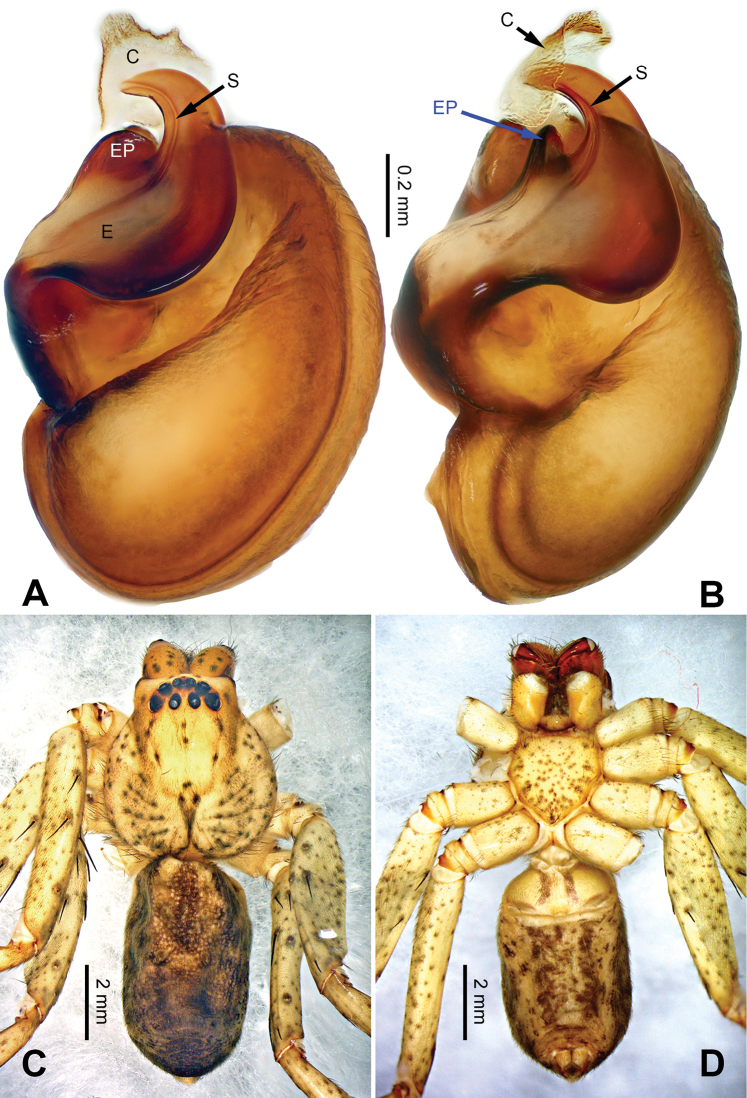
*Pseudopodanyingchiensis* Zhao & Li, sp. n., male holotype. Right bulb horizontally flipped for the sake of comparison. **A** Bulb, ventral view **B** Bulb, dorsal view **C** Habitus, dorsal view **D** Habitus, ventral view. Scale bar equal for **A, B**.

Median-sized *Pseudopoda* species. Male resembles *P.gogona* Jäger, 2001 (see Jäger 2001: 58, figure 36a–e) and *P.gibberosa* Zhang, Zhang & Zhang, 2013 (see Zhang et al. 2013a: 274, figs 1–12) by: embolus sickle-shaped, with blunt embolic projection, tip pointing prolaterally (Figure 17A, B). It can be distinguished by: RTA well developed, divided into dRTA and vRTA, dRTA finger-like, elongated and curved (Figure 16B, C; dRTA distinctly shorter in *P.gogona* and *P.gibberosa*).

###### Description.

Male (holotype). Body length 9.9, DS length 4.8, DS width 4.3, OS length 5.1, OS width 3.3. Eyes: AME 0.19, ALE 0.25, PME 0.20, PLE 0.32, AME-AME 0.20, AME-ALE 0.10, PME-PME 0.28, PME-PLE 0.38, AME-PME 0.38, ALE-PLE 0.34, CH AME 0.31, CH ALE 0.26. Leg formula: II-I-IV-III. Spination: palp 131, 101, 2111; legs: femur I-III 323, IV 331; patella I-III 001, IV 000; tibia I-IV 2026; metatarsus I-II 2024, III 3035, IV 3037. Measurements of palp and legs: palp 7.2 (2.5, 1.1, 1.3, -, 2.3), leg I 23.5 (6.0, 2.5, 6.3, 6.7, 2.0), leg II 25.6 (6.6, 2.6, 7.0, 7.3, 2.1), leg III 21.8 (6.0, 2.3, 5.8, 6.0, 1.7), leg IV 23.4 (6.3, 2.2, 5.9, 7.0, 2.0). Promargin of chelicerae with three teeth, retromargin with four teeth. Cheliceral furrow with ca. 18 denticles.

Palp as in diagnosis. Retrolateral margin of cymbium swollen. RTA arising basally to mesially from tibia, vRTA broad in retrolateral view (Figure 16A–C). Sperm duct running submarginally retrolaterally in tegulum. Embolus arising from tegulum at 9 o’clock position. Conductor arising from tegulum at 12 o’clock position, slightly leaning prolaterally to cover the tip of embolus (Figure 17A, B).

Coloration in ethanol: carapace yellowish. Radial furrows and fovea brown. Dorsal opisthosoma brown. Legs yellowish brown, with randomly distributed dark brown dots (Figure 17C, D).

Female. Unknown.

###### Distribution.

Known only from the type locality.

##### Pseudopoda putaoensis

Taxon classificationAnimaliaAraneaeSparassidae

Zhao & Li sp. n.

http://zoobank.org/068BE24A-D6EB-4B24-B535-537D603F6B17

Figs 18 , 19 , 37

###### Type material.

**Holotype** ♂: Myanmar, Kachin State, Putao, Hponkanrazi Wildlife Sanctuary roadside between Camp 2 to Camp 3, 27°37.150'N, 96°58.917'E, 2806 m, 16 XII 2016, J. Wu.

###### Etymology.

The specific name refers to the type locality; adjective.

###### Diagnosis.

**Figure 18.**
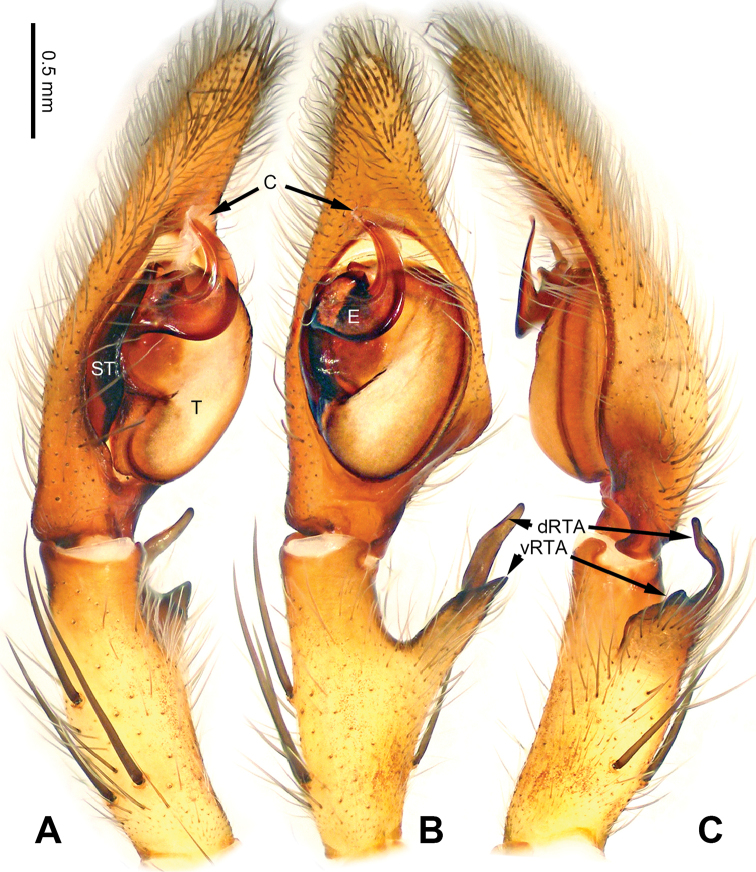
*Pseudopodaputaoensis* Zhao & Li, sp. n., left palp of male holotype. **A** Prolateral view **B** Ventral view **C** Retrolateral view. Scale bar equal for **A, B, C**.

**Figure 19.**
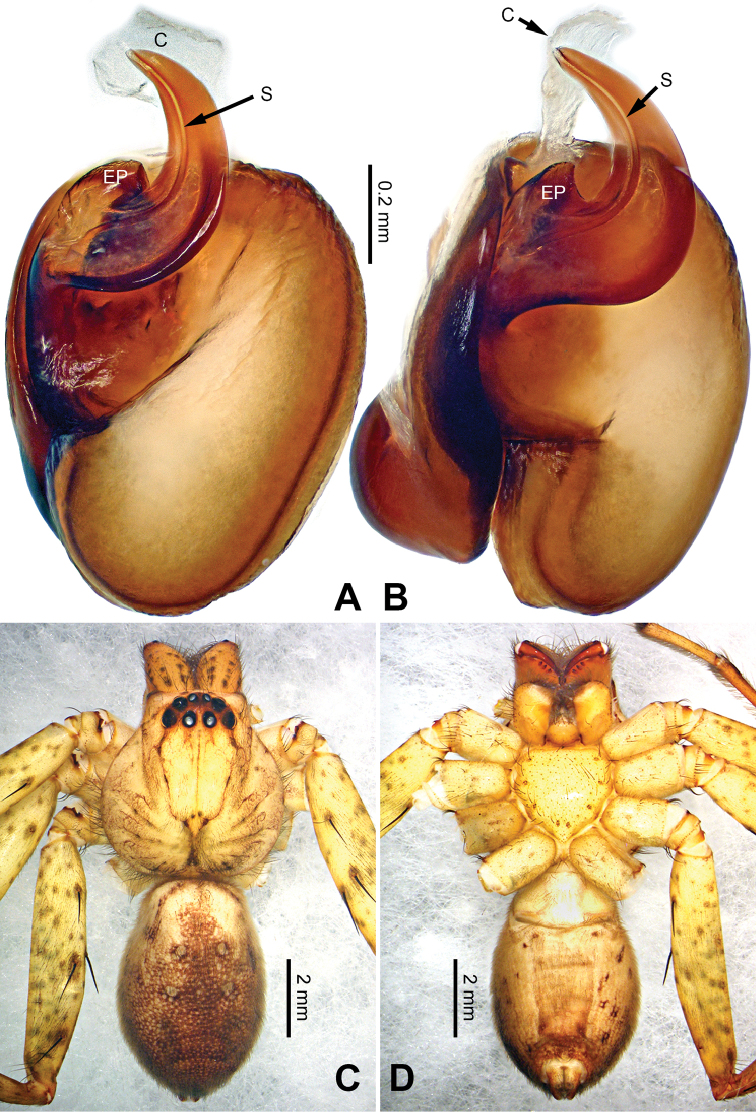
*Pseudopodaputaoensis* Zhao & Li, sp. n., male holotype. **A** Bulb, ventral view **B** Bulb, dorsal view **C** Habitus, dorsal view **D** Habitus, ventral view. Scale bar equal for **A, B**.

Median-sized *Pseudopoda* species. Male resembles *P.platembola* Jäger, 2001 (see Jäger 2001: 57, figure 35a–e), *P.nyingchiensis* Zhao & Li, sp. n. (see Figs 16–17) and *P.huberi* Jäger, 2015 (see Jäger 2015: 346, figs 84–90, 97) by: 1. dRTA finger-like (Figure 18B, C); 2. embolus sickle-shaped (Figure 19A, B). It can be distinguished from the three congeners by the following combination of characters: 1. embolic projection pronounced, emerging from the prolateral margin of embolus (Figure 19A, B; absent in *P.platembola*); 2. cymbium slender and elongated (Figure 18B; shorter and wider in *P.nyingchiensis* Zhao & Li, sp. n. and *P.platembola*); 3. flange absent near the tip of embolus (present in *P.huberi*).

###### Description.

Male (holotype). Body length 9.9, DS length 4.7, DS width 4.1, OS length 5.2, OS width 3.0. Eyes: AME 0.19, ALE 0.31, PME 0.19, PLE 0.31, AME-AME 0.19, AME-ALE 0.12, PME-PME 0.29, PME-PLE 0.38, AME-PME 0.36, ALE-PLE 0.28, CH AME 0.35, CH ALE 0.30. Leg formula: II-I-IV-III. Spination: palp 131, 101, 2111, legs: femur I-II 323, III 322, IV 331; patella I-III 101, IV 000; tibia I-II 2226, III-IV 2126; metatarsus I-II 2024, III 3025, IV 3036. Measurements of palp and legs: palp 7.6 (2.6, 1.3, 1.5, -, 2.2), leg I 24.5 (6.5, 2.3, 6.5, 7.0, 2.2), leg II 26.8 (7.0, 2.6, 7.1, 7.8, 2.3), leg III 22.3 (5.6, 2.2, 6.0, 6.3, 1.9), leg IV 23.8 (6.2, 2.1, 6.1, 7.2, 2.2). Promargin of chelicerae with three teeth, retromargin with four teeth. Cheliceral furrow with ca. 30 denticles.

Palp as in diagnosis. Cymbium elongated, retrolateral bulge present. RTA arising mesially from tibia, vRTA broad and humble in retrolateral view (Figure 18A–C). Sperm duct running submarginally retrolaterally in tegulum. Embolus arising from tegulum at 10 o’clock position. Embolic projection broad and sub-triangular. Conductor arising from tegulum at 12 o’clock position, slightly leaning prolaterally to cover the tip of embolus (Figure 19A, B).

Coloration in ethanol: carapace yellowish. Radial furrows and fovea brown. Dorsal opisthosoma brown. Legs yellowish brown, with randomly distributed dark brown dots (Figure 19C, D).

Female. Unknown.

###### Distribution.

Known only from the type locality.

##### Pseudopoda shacunensis

Taxon classificationAnimaliaAraneaeSparassidae

Zhao & Li sp. n.

http://zoobank.org/A81F9E0F-CD1C-42AC-B2E0-E5D95AC5EC98

Figs 20 , 21 , 37

###### Type material.

**Holotype** ♂: China, Jiangxi Province, Ji’an city, Taihe County, Shacun Town, Chayuan Village, Guangshiyan, 26°31.214'N, 115°06.616'E, 3124 m, 3 V 2013, Y. Luo & J. Liu.

###### Etymology.

The specific name refers to the type locality; adjective.

###### Diagnosis.

**Figure 20.**
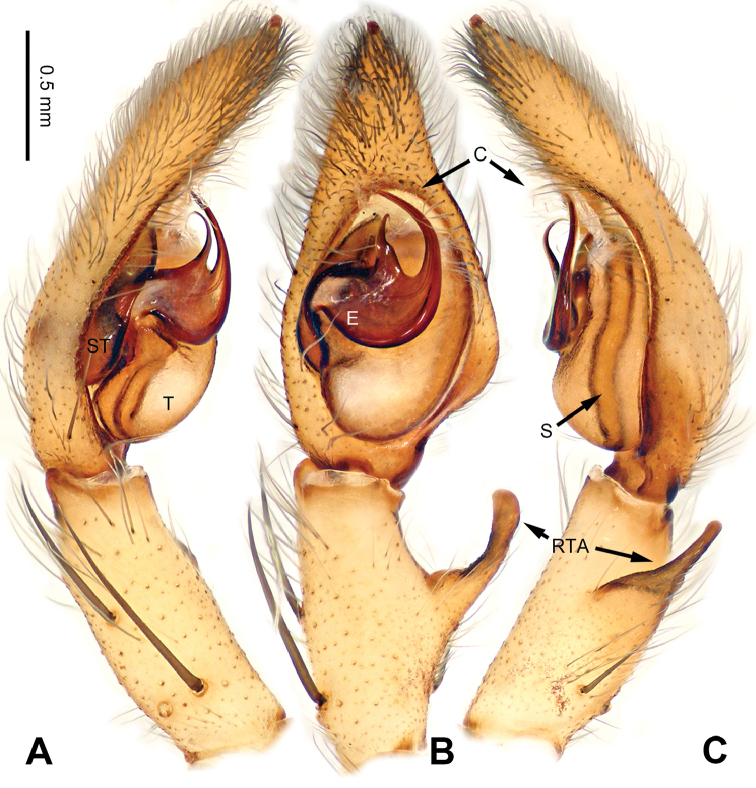
*Pseudopodashacunensis* Zhao & Li, sp. n., left palp of male holotype. **A** Prolateral view **B** Ventral view **C** Retrolateral view. Scale bar equal for **A, B, C**.

**Figure 21.**
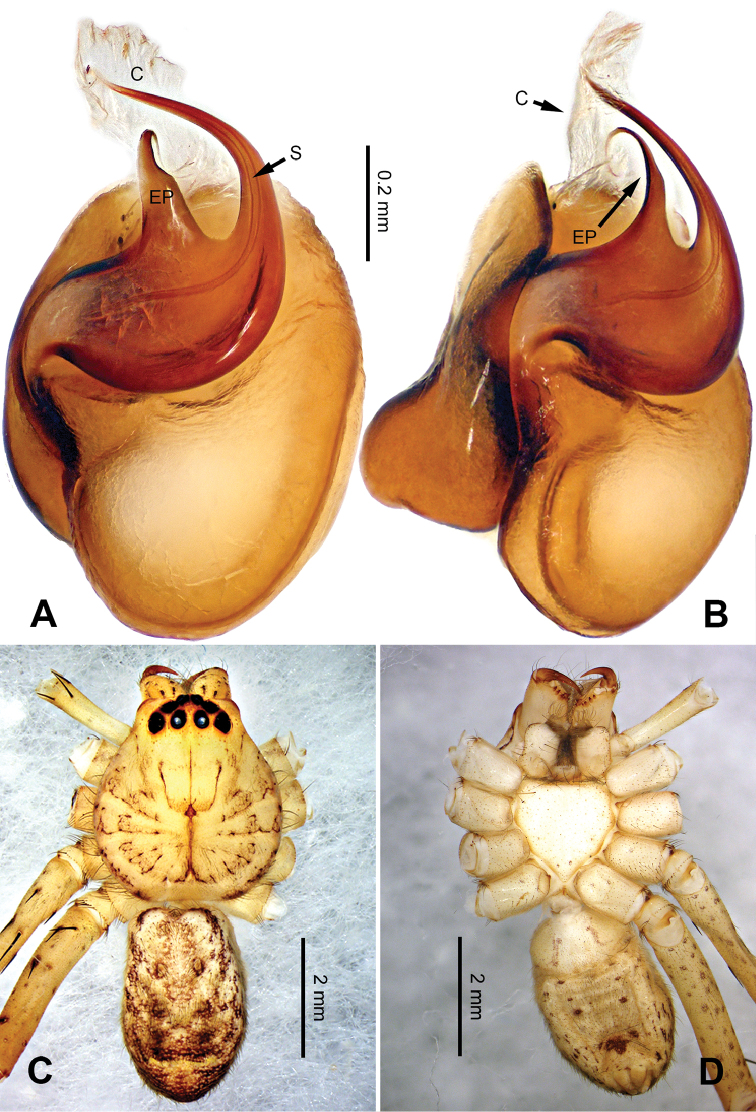
*Pseudopodashacunensis* Zhao & Li, sp. n., male holotype. **A** Bulb, ventral view **B** Bulb, dorsal view **C** Habitus, dorsal view **D** Habitus, ventral view. Scale bar equal for **A, B**.

Small-sized *Pseudopoda* species. Male resembles *P.lushanensis* (Wang, 1990) (see Quan et al. 2014: 559, figs 4A–F, 5A–G), *P.martensi* Jäger, 2001 (see Jäger 2001: 66, figs 3a–h, 39a–l, 84) and *P.hyatti* Jäger, 2001 (see Jäger 2001: 72, figs 41j–m, 84) by: 1. embolus sickle-shaped, its distal part filiform (Figure 21A, B); 2. RTA arising mesially from tibia, single-branched (Figure 20B, C). It can be distinguished by the elongated embolic projection curved backwards dorsally, with its tip ending near the base of conductor (Figure 21A, B; absent in *P.lushanensis*; significantly shorter in *P.hyatti* and *P.martensi*).

###### Description.

Male (holotype). Body length 6.8, DS length 3.4, DS width 3.3, OS length 3.4, OS width 2.5. Eyes: AME 0.20, ALE 0.25, PME 0.20, PLE 0.25, AME-AME 0.18, AME-ALE 0.06, PME-PME 0.24, PME-PLE 0.30, AME-PME 0.31, ALE-PLE 0.27, CH AME 0.30, CH PLE, 0.28. Spination: palp 131, 101, 2111; legs: femur III 323, IV 321; patella III-IV 001; tibia III-IV 2126; metatarsus III 3025, IV 3035. Measurements of palp and legs: palp 5.4 (1.8, 0.8, 1.1, -, 1.7), leg I -, leg II -, leg III 14.3 (4.0, 1.4, 4.0, 3.6, 1.3), leg IV 16.7 (4.3, 1.4, 4.5, 5.0, 1.5). Promargin of chelicerae with three teeth, retromargin with four teeth. Cheliceral furrow with ca. 24 denticles.

Palp as in diagnosis. RTA arising mesially from tibia (Figure 20A–C). Sperm duct running submarginally retrolaterally in tegulum. Embolus arising from tegulum at 9–10 o’clock position with its basal part broadened and its distal part filiform. Embolic projection arising mesially from embolus, steeply narrowed at its distal half. Distal part of embolic projection filiform, curved, and running backwards to the tegulum. Conductor arising from tegulum at 12 o’clock position, leaning prolaterally and covering the tip of embolus (Figure 21A, B).

Coloration in ethanol: carapace yellow. Radial furrows and fovea dark brown. Dorsal opisthosoma bright brown with reddish brown pattern composed of dense reddish brown dots. Legs yellow, with reddish brown dots and patches (Figure 21C, D).

Female. Unknown.

###### Distribution.

Known only from the type locality.

##### Pseudopoda shuo

Taxon classificationAnimaliaAraneaeSparassidae

Zhao & Li sp. n.

http://zoobank.org/2F891F63-2912-4965-B878-B5FB105EE0D2

**Figure 22.**
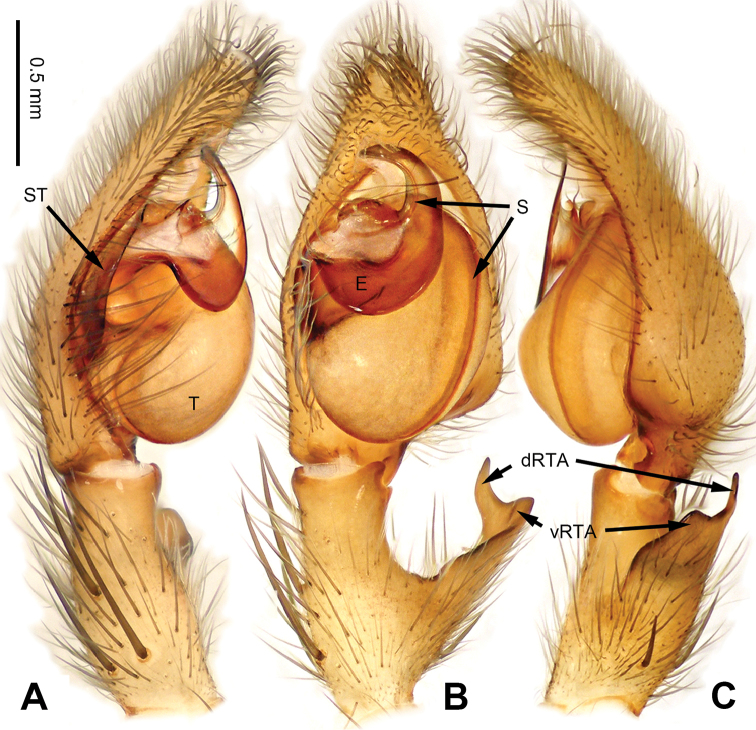
*Pseudopodashuo* Zhao & Li, sp. n., right palp of male holotype, horizontally flipped for the sake of comparison. **A** Prolateral view **B** Ventral view **C** Retrolateral view. Scale bar equal for **A, B, C**.

**Figure 23.**
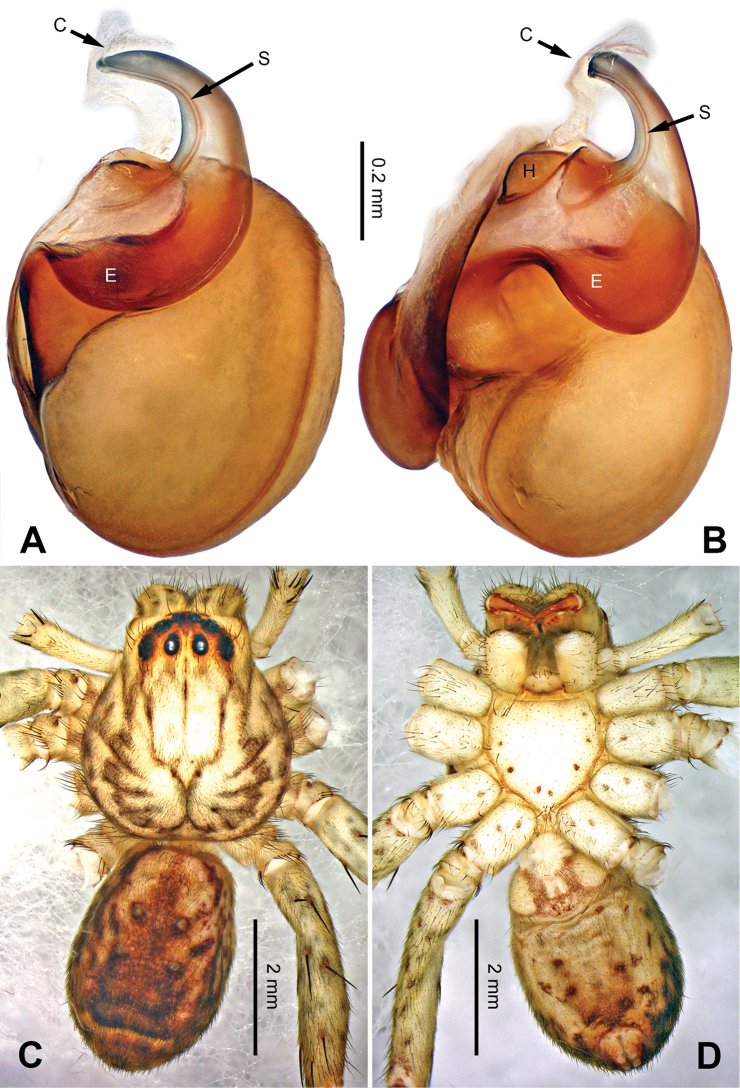
*Pseudopodashuo* Zhao & Li, sp. n., male holotype. Right bulb horizontally flipped for the sake of comparison. **A** Bulb, ventral view **B** Bulb, dorsal view **C** Habitus, dorsal view **D** Habitus, ventral view. Scale bar equal for **A, B**.

**Figure 24.**
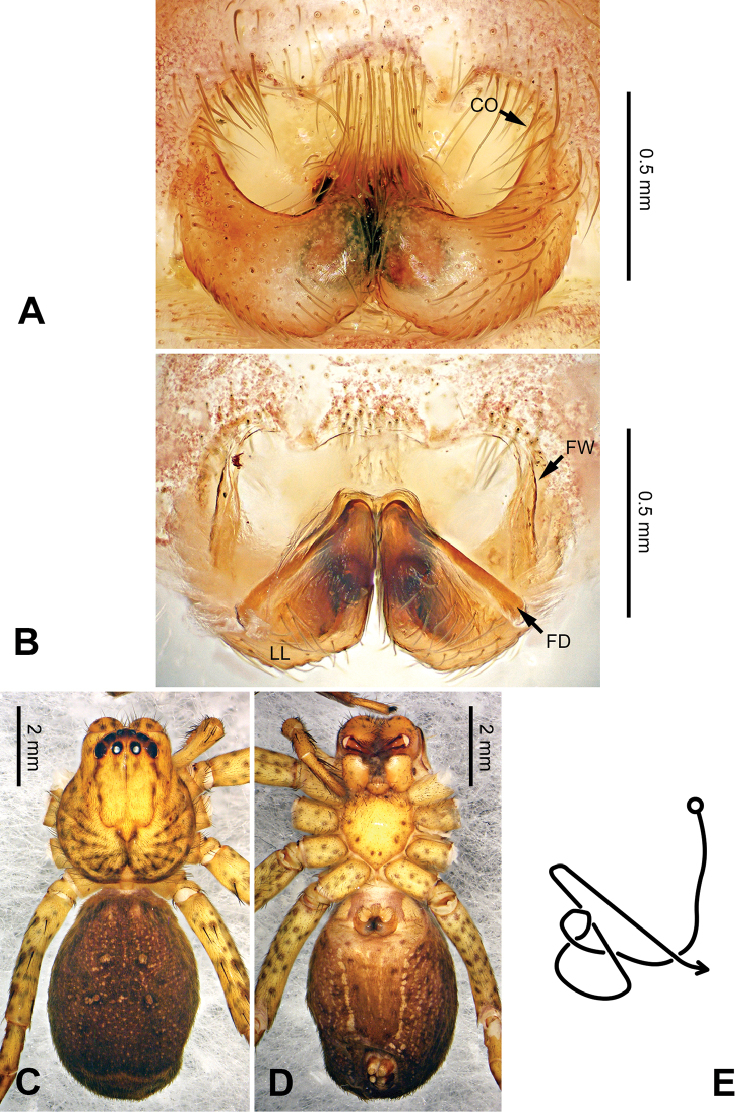
*Pseudopodashuo* Zhao & Li, sp. n., paratype female. **A** Epigyne, ventral view **B** Vulva, dorsal view **C** Habitus, dorsal view **D** Habitus, ventral view **E** Schematic course of internal duct system, dorsal view.

Figs 22 , 23 , 24 , 37

###### Type material.

**Holotype** ♂: China, Tibet Autonomous Region, Nyingchi Prefecture, Medog County, 44 km of the road of Bomi to Medog, 29°42.516'N, 95°34.650'E, 2787 m, 30 VIII 2015, J. Wu. **Paratype**: 1 ♀, same data as holotype.

###### Etymology.

The specific name is derived from the Chinese Pinyin word for ‘gigantism’ (shuò), referring to the relatively larger bulb on male palp than other *Pseudopoda* species; noun in apposition.

###### Diagnosis.

Small-sized *Pseudopoda* species. Male resembles *P.zhangi* Fu & Zhu, 2008 (see Fu and Zhu 2008: 657, figs 1–5), *P.gogona* Jäger, 2001 (see Jäger 2001: 58, figure 36a–e), *P.gibberosa* Zhang, Zhang & Zhang, 2013 (see Zhang et al. 2013a: 274, figs 1–12) and *P.acuminata* Zhang, Zhang & Zhang, 2013 (see Zhang et al. 2013b: 39, figs 1–17) by: 1. tip of embolus sickle-shaped and directing prolaterally (Figure 23A, B); 2. RTA dividing into dRTA and vRTA, dRTA hook-like rather than finger-like (Figure 22B, C). It can be distinguished from the four congeners by the following combination of characters: 1. cymbium shortened, while tegulum swollen, covering a prominently bigger proportion of cymbium in ventral view than in *P.zhangi*, *P.gogona*, and *P.acuminata* (Figure 22B); 2. embolic projection as a small hump on the basal part of embolus (Figure 23A, B; pointed and near the tip of embolus in *P.acuminata*; at the same position but far more distinct in *P.gibberosa*); 3. single hump arising from tegulum near the base of conductor, humble, almost entirely covered by embolus in ventral view (Figure 23A, B; more distinct and clearly visible in ventral view in *P.zhangi*).

Female can be distinguished from other *Pseudopoda* species except *P.contraria* Jäger & Vedel, 2007 (see Jäger and Vedel 2007: 31, figs 114–119) and *P.zhangi* Fu & Zhu, 2008 (see Fu and Zhu 2008: 657, figs 1–5) by: 1. lateral lobes crescent-shaped (Figure 24A, B); 2. internal duct system with loops looming in ventral view as dark shades near the median margin of lateral lobes (Figure 24A); 3. posterior part of first winding of internal duct system hidden in lateral lobes in dorsal view (Figure 24B). It can be distinguished from the two congeners by the following combination of characters: 1. anterior bands poorly developed (Figure 24A; more distinct in *P.contraria*); 2. median margin of lateral lobe intensely curved, extending in the anterior half of epigynal field (Figure 24A, B; moderately curved in *P.zhangi*).

###### Description.

Male (holotype). Body length 6.5, DS length 3.3, DS width 2.9, OS length 3.2, OS width 2.0. Eyes: AME 0.14, ALE 0.25, PME 0.17, PLE 0.22, AME-AME 0.12, AME-ALE 0.03, PME-PME 0.20, PME-PLE 0.30, AME-PME 0.28, ALE-PLE 0.24, CH AME 0.28, CH ALE 0.24. Leg formula: II-IV-I-III. Spination: palp 131, 101, 2111; legs: femur I-III 323, IV 332; patella I-III 001, IV 000; tibia I-IV 2026; metatarsus I-II 2024, III 3025, IV 3037. Measurements of palp and legs: palp - (-, 0.7, 0.9, -, 1.6), leg I 12.5 (3.5, 1.5, 3.3, 3.1, 1.1), leg II 13.1 (3.7, 1.5, 3.3, 3.1, 1.1), leg III 11.7 (3.4, 1.4, 3.0, 2.9, 1.0), leg IV 12.9 (3.6, 1.2, 3.3, 3.5, 1.3). Promargin of chelicerae with three teeth, retromargin with four teeth. Cheliceral furrow with ca. 25 denticles.

Palp as in diagnosis. Cymbium relatively shortened compared to other *Pseudopoda* species. RTA arising basally from tibia (Figure 22A–C). Sperm duct running submarginally and retrolaterally in tegulum. Embolus arising from tegulum at 10–11 o’clock position. Angle between the tip of embolus and the broad part of embolus is ca. 180°. Conductor arising from tegulum at 12 o’clock position (Figure 23A, B).

Coloration in ethanol: carapace bright brown with dark brown lateral bands. Radial furrows and fovea darker. Dorsal opisthosoma reddish brown with black pattern and a bright transverse band in the posterior half. Legs bright brown, with reddish brown patches (Figure 23C, D).

Female (paratype). Body length 8.8, DS length 3.8, DS width 3.3, OS length 5.0, OS width 3.5. Eyes: AME 0.14, ALE 0.24, PME 0.16, PLE 0.30, AME-AME 0.18, AME-ALE 0.21, PME-PME 0.25, PME-PLE 0.30, AME-PME 0.33, ALE-PLE 0.16, CH AME 0.28, CH ALE 0.24. Leg formula: II-IV-I-III. Spination: palp 131, 101, 1014, 2121; legs: femur I-III 323, IV 331; patella I-IV 000; tibia I-III 2026, IV 2025; metatarsus I-II 2024, III 3025, IV 3037. Measurements of palp and legs: palp 4.1 (1.5, 0.5, 0.7, -, 1.4), leg I 11.5 (3.4, 1.5, 3.0, 2.6, 1.0), leg II 12.2 (3.6, 1.6, 3.2, 2.8, 1.0), leg III 10.6 (3.2, 1.3, 2.8, 2.4, 0.9), leg IV 11.9 (3.5, 1.3, 2.9, 3.1, 1.1). Promargin of chelicerae with three teeth, retromargin with four teeth. Cheliceral furrow with ca. 28 denticles.

Epigyne as in diagnosis. Epigynal field longer in transverse axis, with poorly developed anterior bands and trilobate anterior margin. Lateral lobes longer in transverse axis, curved. Median margin of lateral lobe converged, with the posterior part V-shaped. Posterior incision of lateral lobe indistinct or absent (Figure 24A, B).

Coloration in ethanol: as in male, but generally darker. Ventral opisthosoma with a pair of bright, longitudinal, dashed lines (Figure 24C, D).

###### Distribution.

Known only from the type locality.

##### Pseudopoda subbirmanica

Taxon classificationAnimaliaAraneaeSparassidae

Zhao & Li sp. n.

http://zoobank.org/0B4CC01D-0EC4-4F4B-997B-B44E75B53DC1

**Figure 25.**
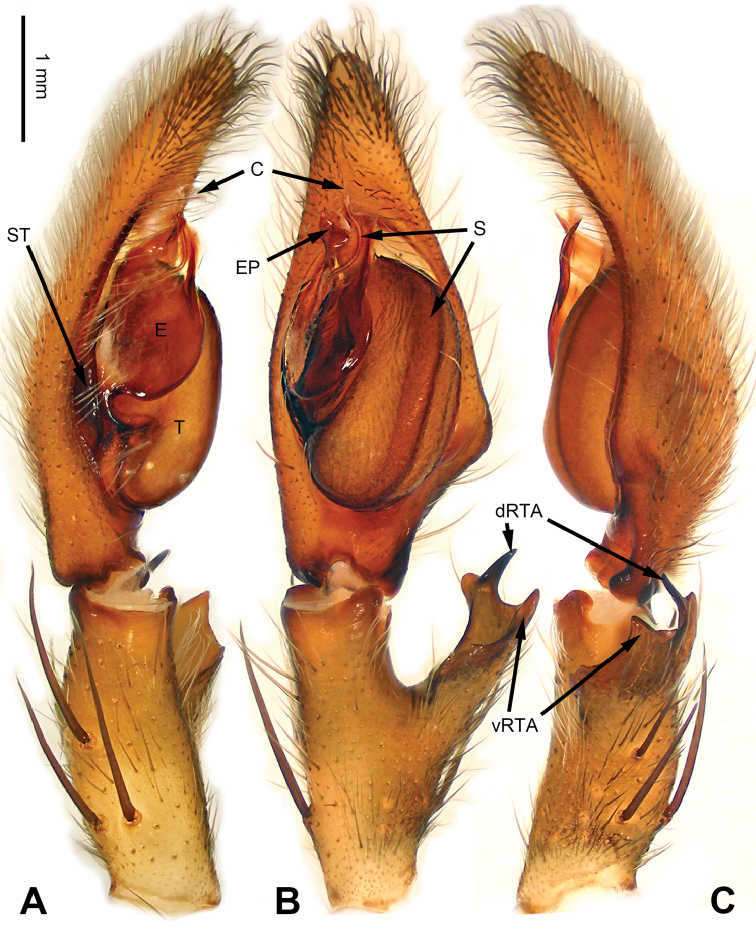
*Pseudopodasubbirmanica* Zhao & Li, sp. n., left palp of male holotype. **A** Prolateral view **B** Ventral view **C** Retrolateral view. Scale bar equal for **A, B, C**.

**Figure 26.**
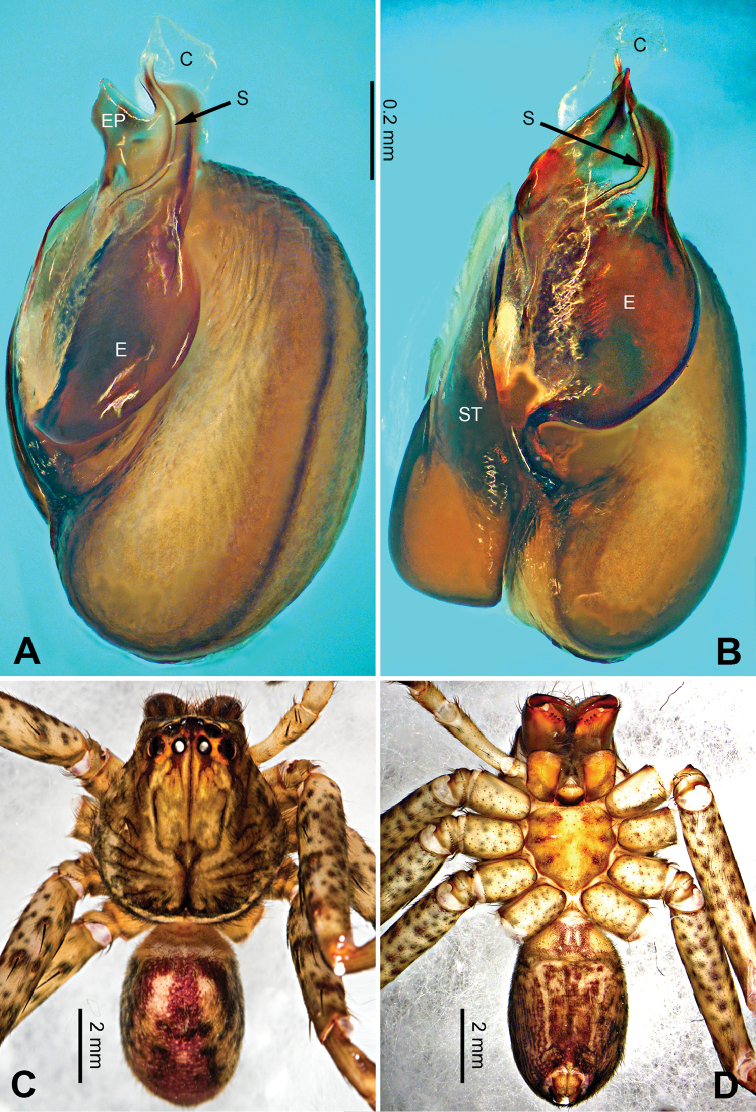
*Pseudopodasubbirmanica* Zhao & Li, sp. n., male holotype. **A** Bulb, ventral view **B** Bulb, dorsal view **C** Habitus, dorsal view **D** Habitus, ventral view. Scale bar equal for **A, B**.

**Figure 27.**
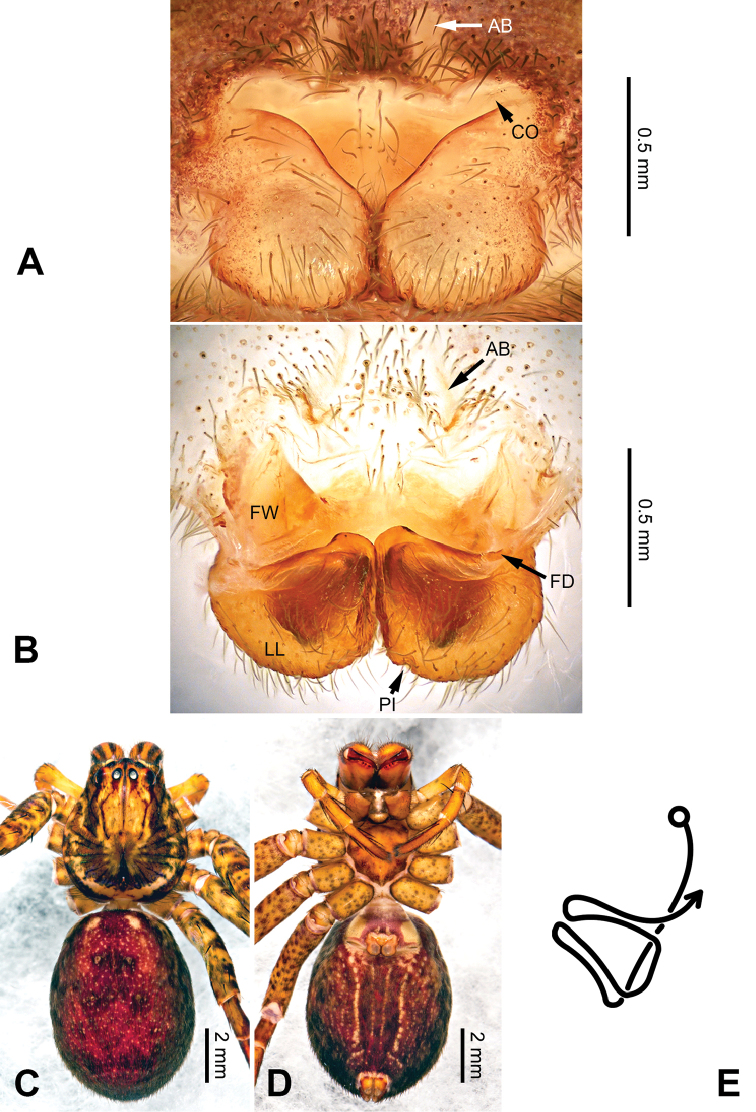
*Pseudopodasubbirmanica* Zhao & Li, sp. n., paratype female. **A** Epigyne, ventral view **B** Vulva, dorsal view **C** Habitus, dorsal view **D** Habitus, ventral view **E** Schematic course of internal duct system, dorsal view.

Figs 25 , 26 , 27 , 37

###### Type material.

**Holotype** ♂: Myanmar, Kachin State, Putao, Hponkanrazi Wildlife Sanctuary roadside between Camp 1 to Camp 2, 27°36.550'N, 96°58.850'E, 2252 m, 17 XII 2016, J. Wu. **Paratypes**: 1 ♂, same locality as holotype, 14 V 2017, Z. Chen & J. Wu; 1 ♀, same locality as holotype, 18 V 2017.

###### Etymology.

The specific name refers to the similarity of its female individual to *P.birmanica* Jäger, 2001; adjective.

###### Diagnosis.

Small to median-sized *Pseudopoda* species. Male resembles *P.digitata* Jäger & Vedel, 2007 (see Jäger and Vedel 2007: 29, figs 105–113) by: embolus with prolateral projection near the tip (Figure 26A, B). It can be distinguished from the latter by the following combination of characters: 1. tip of embolus pointed (Figure 26A, B; broad and blunt in *P.digitata*); 2. dRTA with a prolateral protrusion (Figure 25B, C).

Female extremely resembles *P.birmanica* Jäger, 2001 (see Jäger 2001: 75, figure 43a–c) with slight differences in their internal duct systems. For example, the female of *P.subbirmanica* Zhao & Li, sp. n. lacks an anterior loop near the fertilization duct, which is present in *P.birmanica* (Figure 27B, E).

###### Description.

Male (holotype). Body length 9.3, DS length 5, DS width 4.5, OS length 4.3, OS width 3.0. Eyes: AME 0.16, ALE 0.33, PME 0.24, PLE 0.38, AME-AME 0.25, AME-ALE 0.13, PME-PME 0.24, PME-PLE 0.40, AME-PME 0.44, ALE-PLE 0.40, CH AME 0.48, CH ALE 0.37. Leg formula: IV-II-I-III. Spination: palp 131, 101, 2111; legs: femur I-III 323, IV 321; patella I-IV 001; tibia I-IV 2026; metatarsus I-II 1014, III 3035, IV 3037. Measurements of palp and legs: palp 6.9 (2.3, 1.1, 1.3, -, 2.2), leg I 20.3 (5.8, 2.0, 5.6, 5.3, 1.6), leg II 20.5 (5.9, 2.0, 5.8, 5.1, 1.7), leg III 18.6 (5.4, 2.0, 5.0, 4.6, 1.6), leg IV 20.6 (6.0, 1.8, 5.4, 5.4, 2.0). Promargin of chelicerae with three teeth, retromargin with four teeth. Cheliceral furrow with ca. 20 denticles.

Palp as in diagnosis. Cymbium slender. RTA arising mesially from tibia (Figure 25A–C). Sperm duct running submarginally retrolaterally in tegulum. Embolus broad and nearly sickle-shaped, arising from tegulum at 9 o’clock position. Tip of embolus tapering and bending slightly. Conductor arising from tegulum at 12 o’clock position (Figure 26A, B).

Coloration in ethanol: carapace yellowish brown. Radial furrows and fovea dark brown. Dorsal opisthosoma reddish brown. Ventral opisthosoma with a pair of light transverse bands. Legs yellowish brown, with randomly distributed reddish brown dots (Figure 26C, D).

Female (paratype). Body length 12.2, DS length 5.1, DS width 4.8, OS length 7.1, OS width 5.1. Eyes: AME 0.16, ALE 0.29, PME 0.26, PLE 0.34, AME-AME 0.19, AME-ALE 0.08, PME-PME 0.26, PME-PLE 0.44, AME-PME 0.46, ALE-PLE 0.32, CH AME 0.36, CH ALE 0.30. Leg formula: II-I-IV-III. Spination: palp 131, 101, 2121, 1014; legs: femur I-II 323, III 322, IV 331; patella I-IV 001; tibia I-IV 2026; metatarsus I-II 1014, III 3025, IV 3037. Measurements of palp and legs: palp 6.1 (1.8, 1.1, 1.2, -, 2.0), leg I 15.4 (4.3, 2.0, 4.1, 3.6, 1.4), leg II 16.1 (4.5, 1.9, 4.3, 3.8, 1.6), leg III 14.1 (4.3, 1.8, 3.4, 3.2, 1.4), leg IV 14.8 (4.1, 1.6, 3.6, 4.0, 1.5). Promargin of chelicerae with three teeth, retromargin with four teeth. Cheliceral furrow with ca. 25 denticles.

Epigyne as in diagnosis. Epigynal field longer in transverse axis. Anterior bands distinct, anterior margin slightly trilobate. Lateral lobes longer in transverse axis. Median margin of lateral lobes converged on the central axis, with anterior part V-shaped. Anterior margin of lateral lobe directed forward and then laterally (Figure 27A). Half of first winding of internal duct system hidden behind lateral lobe in dorsal view (Figure 27B). Loops of internal duct system (spermatheca) sub-triangular (Figure 27B, E).

Coloration in ethanol: as in male, but generally darker. Carapace with dark pattern (Figure 27C, D).

###### Distribution.

Known only from the type locality.

##### Pseudopoda titan

Taxon classificationAnimaliaAraneaeSparassidae

Zhao & Li sp. n.

http://zoobank.org/D3CCBE41-AE88-4583-9BE6-4EC20DEA3366

**Figure 28.**
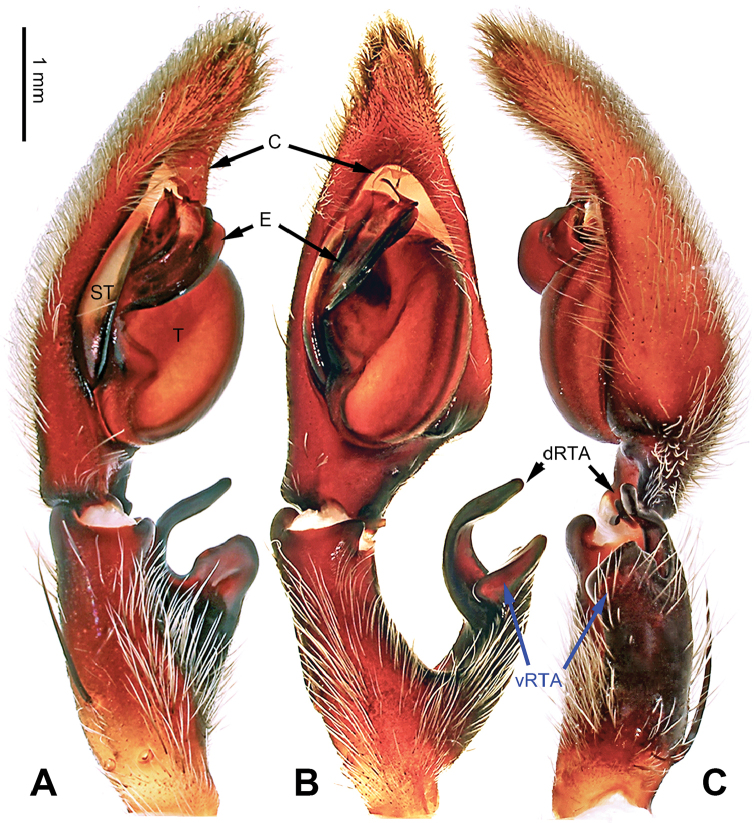
*Pseudopodatitan* Zhao & Li, sp. n., left palp of male holotype. **A** Prolateral view **B** Ventral view **C** Retrolateral view. Scale bar equal for **A, B, C**.

**Figure 29.**
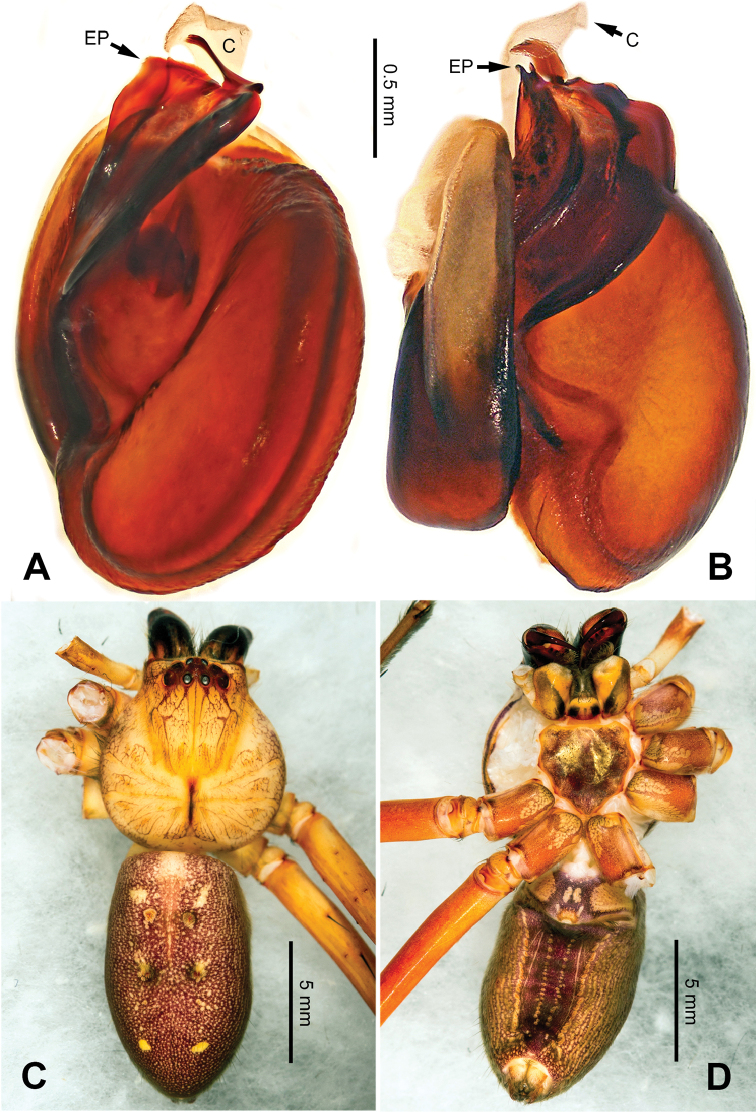
*Pseudopodatitan* Zhao & Li, sp. n., male holotype. **A** Bulb, ventral view **B** Bulb, dorsal view **C** Habitus, dorsal view **D** Habitus, ventral view. Scale bar equal for **A, B**.

**Figure 30.**
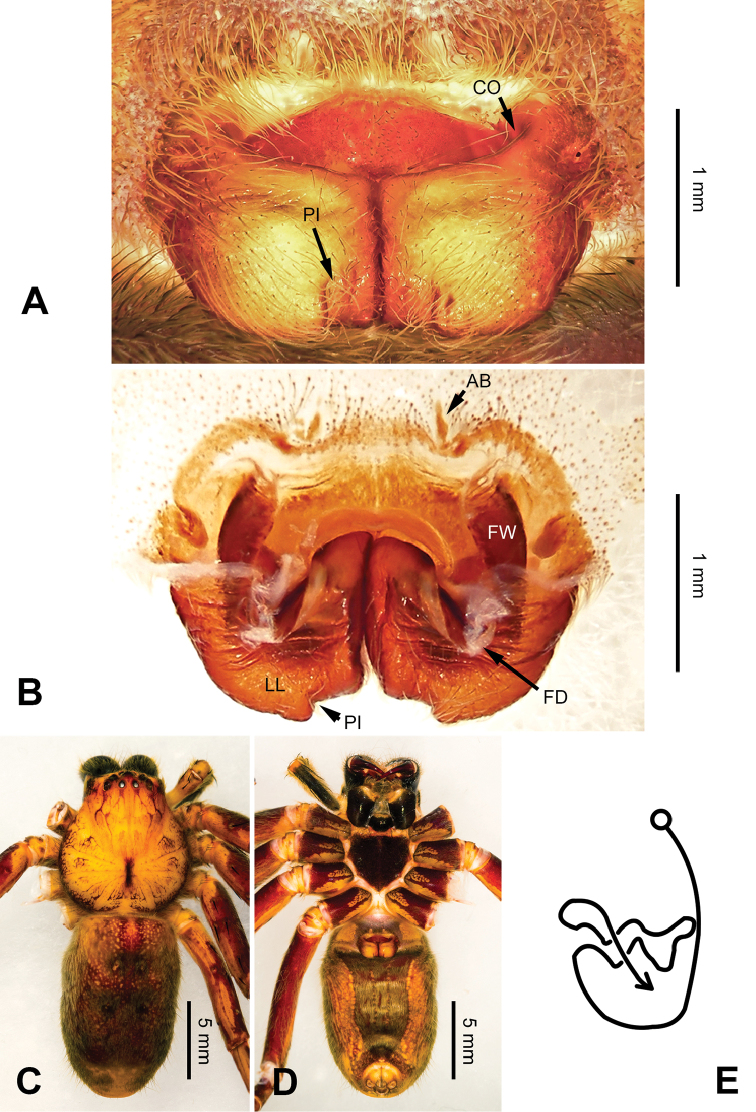
*Pseudopodatitan* Zhao & Li, sp. n., paratype female. **A** Epigyne, ventral view **B** Vulva, dorsal view **C** Habitus, dorsal view **D** Habitus, ventral view **E** Schematic course of internal duct system, dorsal view.

Figs 28 , 29 , 30 , 37

###### Type material.

**Holotype** ♂: Myanmar, Kachin State, Putao, Hponkanrazi Wildlife Sanctuary, roadside between Camp 2 to Camp 3, 27°36.867'N, 96°58.933'E, 2491 m, 15 XII 2016, J. Wu. **Paratype**: 1 ♀, same locality as holotype, 12 V 2017, J. Wu & Z. Chen.

###### Etymology.

The specific name is derived from the name of giants in Greek myth, referring to the gigantic size of this species; noun in apposition.

###### Diagnosis.

Large-sized *Pseudopoda* species. Male resembles *P.emei* Zhang, Zhang & Zhang, 2013 (see Zhang et al. 2013b: 44, figs 18–33), *P.namkhan* Jäger, Pathoumthong & Vedel, 2006 (see Jäger et al. 2006: 222, figs 20–28, 35–40) and *P.mediana* Quan, Zhong & Liu, 2014 (see Quan et al. 2014: 562, figs 6A–C, 7A–C, 8A–D, 9A–C) by: tip of embolus sharply curved and pointing prolaterally (Figure 29A, B). It can be distinguished from the three congeners by the following combination of characters: 1. dRTA well developed, curved, and finger-like (Figure 28A–C; straight and significantly shorter in *P.emei* and *P.mediana*; broadened in *P.namkhan*); 2. tip of embolus slightly broadened (Figure 29B; filiform in *P.emei*); 3. significantly larger in body size.

Female resembles those of *P.gemina* Jäger, Pathoumthong & Vedel, 2006 (see Jäger et al. 2006: 222, figs 14–19, 33–34) and *P.recta* Jäger & Ono, 2001 (see Jäger and Ono 2001: 25, figs 17–22) by: 1. median margin of lateral lobe converged (Figure 30A); 2. slender loops of internal duct system running transversally (Figure 30E). It can be distinguished from the two congeners by the following combination of characters: 1. posterior incisions of lateral lobes distinct (Figure 30A, B; absent in *P.recta* and *P.gemina*); 2. converging part of anterior margins of lateral lobes T-shaped (Figure 30A).

###### Description.

Male (holotype). Body length 19.0, DS length 9.0, DS width 8.0, OS length 10.0, OS width 6.5. Eyes: AME 0.29, ALE 0.38, PME 0.33, PLE 0.38, AME-AME 0.30, AME-ALE 0.13, PME-PME 0.38, PME-PLE 0.60, AME-PME 0.46, ALE-ALE 0.38, CH AME 0.31, CH ALE 0.38. Leg formula: II-I-IV-III. Spination: palp 131, 101, 3100; legs: femur I-III 323, IV 321; patella I-II 101, III-IV 100; tibia I-III 2226, IV 2126; metatarsus I-II 1014, III 2025, IV 2424. Measurements of palp and legs: palp 14.4 (5.1, 2.1, 2.8, -, 4.5), leg I 48.2 (11.5, 4.5, 13.5, 14.0, 4.7), leg II 52.1 (13.5, 4.7, 14.0, 15.0, 4.7), leg III 37.6 (11, 3.7, 10.0, 9.5, 3.4), leg IV 40.6 (11.0, 3.6, 11.0, 11.0, 4.0). Promargin of chelicerae with three teeth, retromargin with four teeth, cheliceral furrow with ca. 30 denticles.

Palp as in diagnosis. Cymbium slender, with distinct retrolateral bulge beside bulb. RTA arising basally from tibia (Figure 28A–C). Sperm duct running submarginally retrolaterally in tegulum. Embolus arising from tegulum at 10 o’clock position, broad, almost straight in ventral view. Tip of embolus leaf-like, sharply curved, and pointing prolaterally. Embolic projection present as two additional triangular rims near the tip. Conductor arising from the tegulum at 12 to 1 o’clock position (Figure 29A, B).

Coloration in ethanol: carapace yellowish brown. Radial furrows and fovea dark brown. Dorsal opisthosoma reddish brown, with white dots and yellow patches. Legs orange. Ventral opisthosoma with two pairs of longitudinal lines composed of orange dots (Figure 29C, D).

Female (paratype). Body length 19.0, DS length 9.0, DS width 8.0, OS length 10.0, OS width 6.5. Eyes: AME 0.40, ALE 0.43, PME 0.30, PLE 0.43, AME-AME 0.34, AME-ALE 0.19, PME-PME 0.46, PME-PLE 0.68, AME-PME 0.53, ALE-PLE 0.47, CH AME 0.47, CH ALE 0.47. Leg formula: II-I-IV-III. Spination: palp 131, 101, 3110, 2020; legs: femur I-III 323, IV 321; patella I-II 101, III-IV 100; tibia I-II 2226, III-IV 2126; metatarsus I-II 1014, III 2024, IV 2037. Measurements of palp and legs: palp 12.6 (4.0, 2.0, 2.6, -, 4.0), leg I 37.5 (11.5, 4.1, 10.0, 9.0, 2.9), leg II 40.2 (11.5, 4.2, 11.5, 10.0, 3.0), leg III 29.1 (8.5, 3.3, 8.0, 6.5, 2.8), leg IV 30.1 (9.0, 3.0, 8.0, 7.5, 2.6). Promargin of chelicerae with three teeth, retromargin with four teeth. Cheliceral furrow with ca. 30 denticles.

Epigyne as in diagnosis. Epigynal field longer in transverse axis, with distinct anterior bands and trilobate anterior margin. Lateral lobes longer in transverse axis, sub-rectangular but narrower laterally. Posterior incision of lateral lobe distinct, near the posterior meeting point of lateral lobes (Figure 30A). Lateral loops of internal duct system running transversally, covered by first winding in dorsal view (Figure 30B, E).

Coloration in ethanol: as in male but generally darker (Figure 30C, D).

###### Distribution.

Known only from the type locality.

##### Pseudopoda xia

Taxon classificationAnimaliaAraneaeSparassidae

Zhao & Li sp. n.

http://zoobank.org/0BDB0064-B929-45F0-A6B8-A0BD071F6F56

Figs 31 , 32 , 37

###### Type material.

**Holotype** ♂: Myanmar, Kachin State, Putao, around Ziradum Village, 27°33.465'N, 97°06.580'E, 1051 m, 8 V 2017, J. Wu & Z. Chen.

###### Etymology.

The specific name is derived from the Chinese Pinyin word ‘jimpness’ (xiá), referring to the narrow abdomen of this species; noun in apposition.

###### Diagnosis.

**Figure 31.**
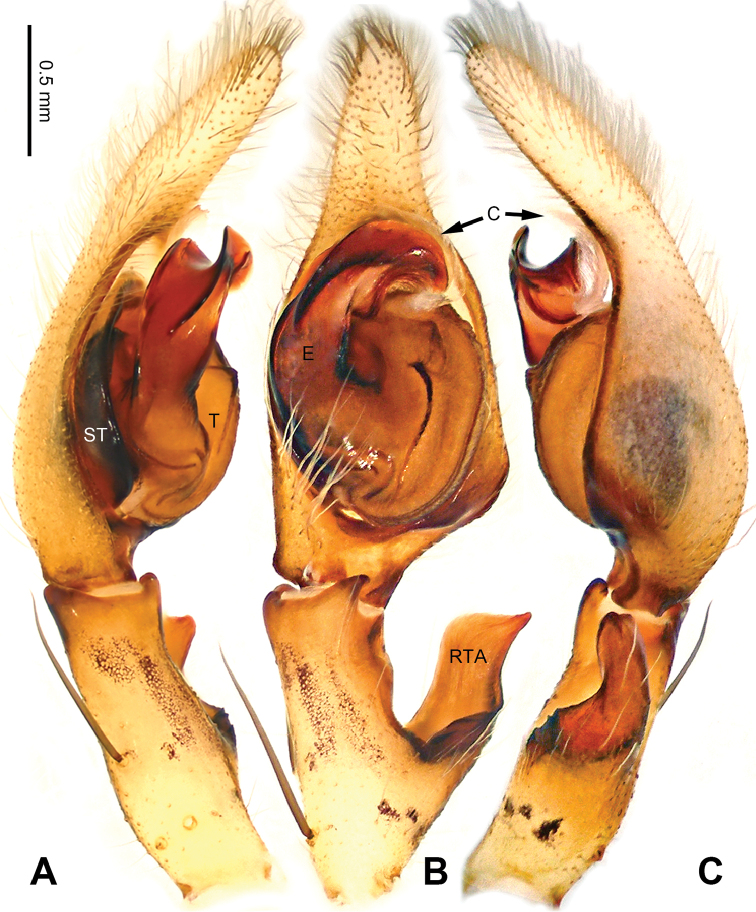
*Pseudopodaxia* Zhao & Li, sp. n., left palp of male holotype. **A** Prolateral view **B** Ventral view **C** Retrolateral view. Scale bar equal for **A, B, C**.

**Figure 32.**
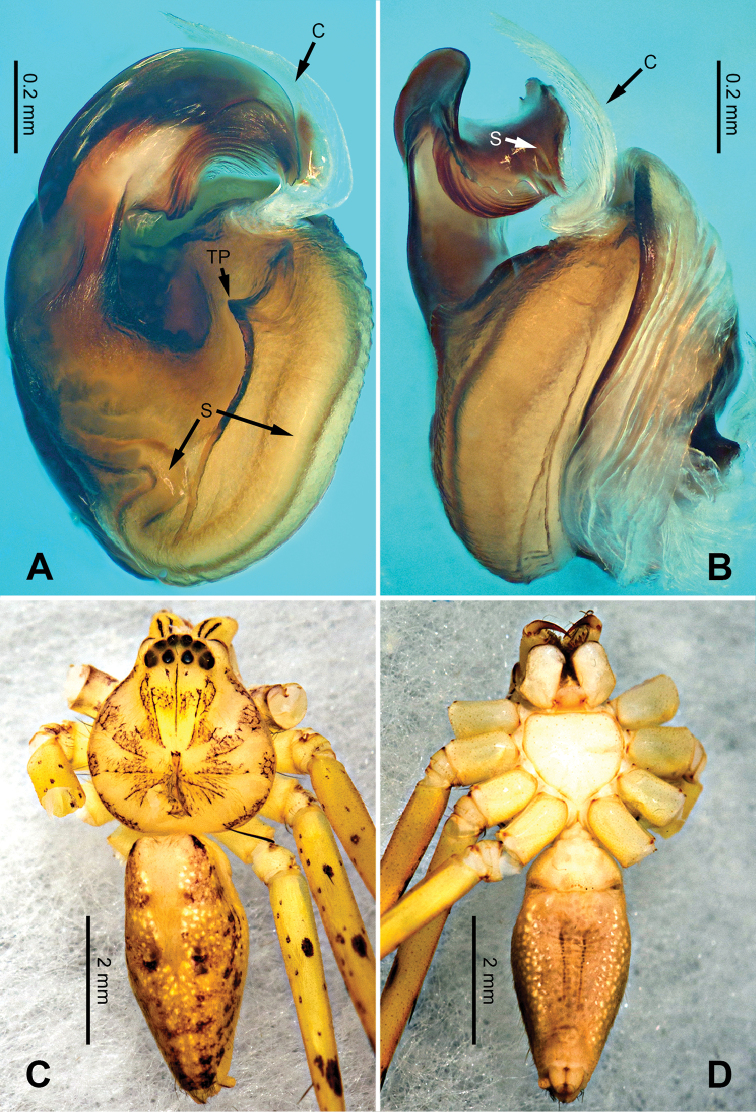
*Pseudopodaxia* Zhao & Li, sp. n., male holotype. **A** Bulb, ventral view **B** Bulb, dorsal view **C** Habitus, dorsal view **D** Habitus, ventral view.

Small-sized *Pseudopoda* species. Male resembles *P.brauni* Jäger, 2001 (see Jäger 2001: 44, figs 26d–g, 27a–d), *P.trisuliensis* Jäger, 2001 (see Jäger 2001: 42, figure 28f–j), *P.prompta* (O. Pickard-Cambridge, 1885) (see Jäger 2000: 63, figs 1–15) and *P.confusa* Jäger, Pathoumthong & Vedel, 2006 (see Jäger et al. 2006: 220, figs 1–13, 29–32) by: embolus running near the prolateral margin of tegulum in ventral view. It can be distinguished from the four congeners by the following combination of characters: 1. RTA simple, with only one apex (Figure 31B, C; RTA with two apices in *P.confusa*); 2. tegulum with a distinct sub-triangular protrusion near the retrolateral margin (Figure 32A; absent in *P.prompta* and *P.confusa*; a blunt hump present on tegulum near the basal part of embolus in *P.trisuliensis* and *P.brauni*); 3. embolus with an extra rim running along the distal part of it (Figure 32B; absent or indistinct in *P.prompta* and *P.confusa*).

###### Description.

Male (holotype). Body length 7.6, DS length 3.1, DS width 3.2, OS length 4.5, OS width 2.2. Eyes: AME 0.15, ALE 0.19, PME 0.15, PLE 0.21, AME-AME 0.12, AME-ALE 0.06, PME-PME 0.14, PME-PLE 0.25, AME-PME 0.21, ALE-PLE 0.24, CH AME 0.16, CH ALE 0.10. Leg formula: I-II-IV-III. Spination: palp 131, 101, 2101; legs: femur I-III 323, IV 322; patella I-II 101, III-IV 001; tibia I 2226, II-III 2116, IV 2126; metatarsus I 1014, II 0014, III 2024, IV 3026. Measurements of palp and legs: palp 5.8 (1.9, 0.9, 1.0, -, 2.0), leg I 28.6 (7.5, 1.8, 8.2, 8.5, 2.6), leg II 26.3 (7.5, .18, 7.0, 7.5, 2.5), leg III 19.1 (5.5, 1.3, 5.2, 5.5, 1.6), leg IV 25.5 (7.0, 2.0, 6.8, 7.5, 2.2). Promargin of chelicerae with three teeth, retromargin with four teeth. Cheliceral furrow with ca. 15 denticles.

Palp as in diagnosis. Cymbium slender, slightly elongated distally. RTA arising basally from tibia (Figure 31A–C). Tegulum with an additional ridge emerging basally, and running distally, ending with a sub-triangular protrusion pointing at the basal part of embolus. Sperm duct running submarginally retrolaterally in tegulum, visible near the base of embolus as an S-shaped duct. Embolus arising from tegulum at 9 to 10 o’clock position. Wrinkles present below the distal part on embolus. Tip of embolus with indention. Conductor arising from tegulum at 1 to 2 o’clock position, slender, bent basally and then directed prolaterally (Figure 32A, B).

Coloration in ethanol: carapace yellow. Radial furrows and fovea black. Dorsal opisthosoma orange, with black pattern and white dots. Ventral opisthosoma with a pair of longitudinal white bands. Legs yellow to orange, with randomly distributed black dots and patches (Figure 32C, D).

Female. Unknown.

###### Distribution.

Known only from the type locality.

##### Pseudopoda yuanjiangensis

Taxon classificationAnimaliaAraneaeSparassidae

Zhao & Li sp. n.

http://zoobank.org/DD1ABF58-C8DB-4E7C-AEC3-B66ADC60EF51

Figs 33 , 37

###### Type material.

**Holotype** ♀: China, Yunnan Province, Yuxi City, Yuanjiang County, Yangchajie Village Nature Reserve, 23°39.632'N, 101°45.564'E, 2114 m, 4 VI 2015, Z. Chen & F. Li.

###### Etymology.

The specific name refers to the type locality; adjective.

###### Diagnosis.

**Figure 33.**
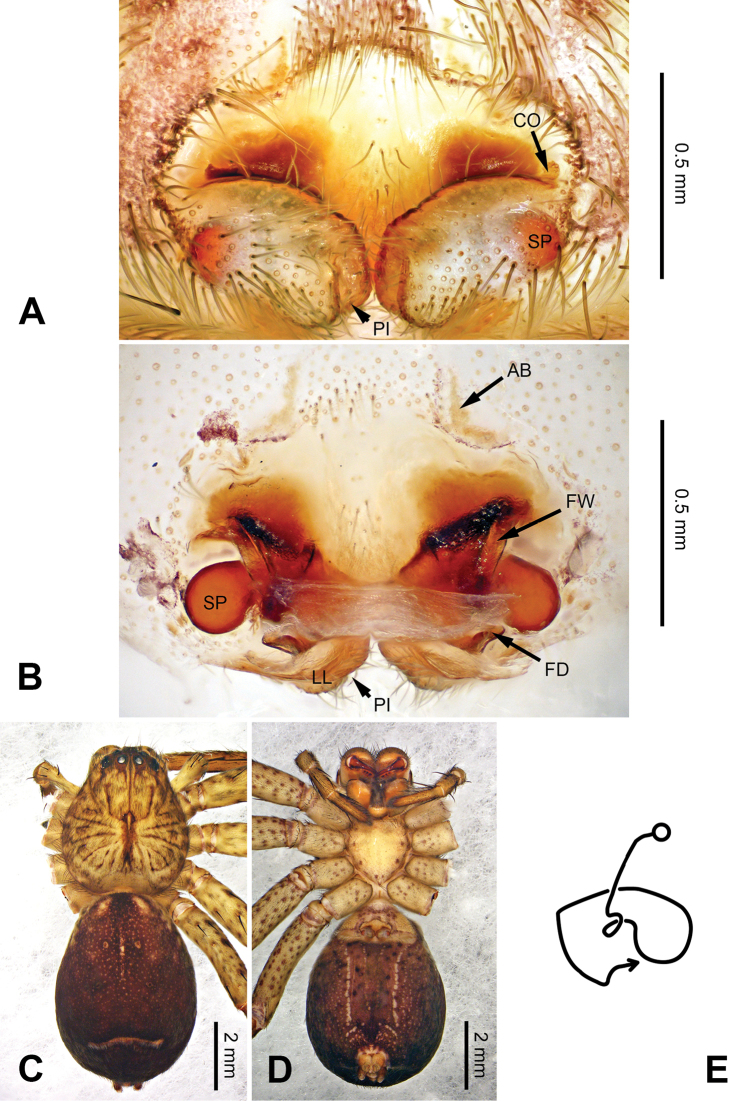
*Pseudopodayuanjiangensis* Zhao & Li, sp. n., female holotype. **A** Epigyne, ventral view **B** Vulva, dorsal view **C** Habitus, dorsal view **D** Habitus, ventral view **E** Schematic course of internal duct system, dorsal view.

Small to median-sized *Pseudopoda* species. Female resembles *P.bibulba* (Xu & Yin, 2000) (see Jäger and Vedel 2007: 15, figs 44–59) by: internal duct system with distinct lateral loops visible through cuticle in ventral view as rounded patches (Figure 33A). It can be distinguished from the latter species by the following combination of characters: 1. anterior bands distinct (Figure 33A; absent in *P.bibulba*); 2. lateral lobes much longer in transverse axis, with anterior margins bending posteriolaterally (Figure 33A; anterior margins bending anteriolaterally and then directed medially in *P.bibulba*).

###### Description.

Female (holotype). Body length 8.5, DS length 2.8, DS width 2.7, OS length 5.7, OS width 4.0. Eyes: AME 0.18, ALE 0.28, PME 0.21, PLE 0.32, AME-AME 0.19, AME-ALE 0.11, PME-PME 0.28, PME-PLE 0.37, AME-PME 0.40, ALE-PLE 0.31, CH AME 0.35, CH ALE 0.30. Leg formula: II-I-IV-III. Spination: palp 131, 101, 2121, 1004; legs: femur I-II 323, III 322, IV 331; patella I-IV 001; tibia I-III 2026, IV 2025; metatarsus I-II 2024, III 3025, IV 3037. Measurements of palp and legs: palp 5.8 (1.7, 1.0, 1.3, -, 1.8), leg I 13.9 (4.0, 1.9, 3.5, 3.2, 1.3), leg II 15.2 (4.3, 2.1, 4.0, 3.4, 1.4), leg III 12.3 (3.7, 1.6, 3.1, 2.8, 1.1), leg IV 13.4 (4.1, 1.6, 3.3, 3.2, 1.2). Promargin of chelicerae with three teeth, retromargin with four teeth. Cheliceral furrow with ca. 38 denticles.

Epigyne as in diagnosis. Epigynal field longer in transverse axis, with anterior bands and trilobate anterior margin. Lateral lobes slightly converged on the central axis. Posterior incision of lateral lobe distinct, near the meeting point of lateral lobes. (Figure 33A, B).

Coloration in ethanol: carapace yellowish brown. Radial furrows and fovea dark brown. Dorsal opisthosoma reddish brown, with a bright transverse band in the posterior half. Legs yellowish brown, with randomly distributed reddish brown dots (Figure 33C, D).

Male. Unknown.

###### Distribution.

Known only from the type locality.

##### Pseudopoda zixiensis

Taxon classificationAnimaliaAraneaeSparassidae

Zhao & Li sp. n.

http://zoobank.org/81384BB2-DF83-472F-B7ED-82BC432366F9

**Figure 34.**
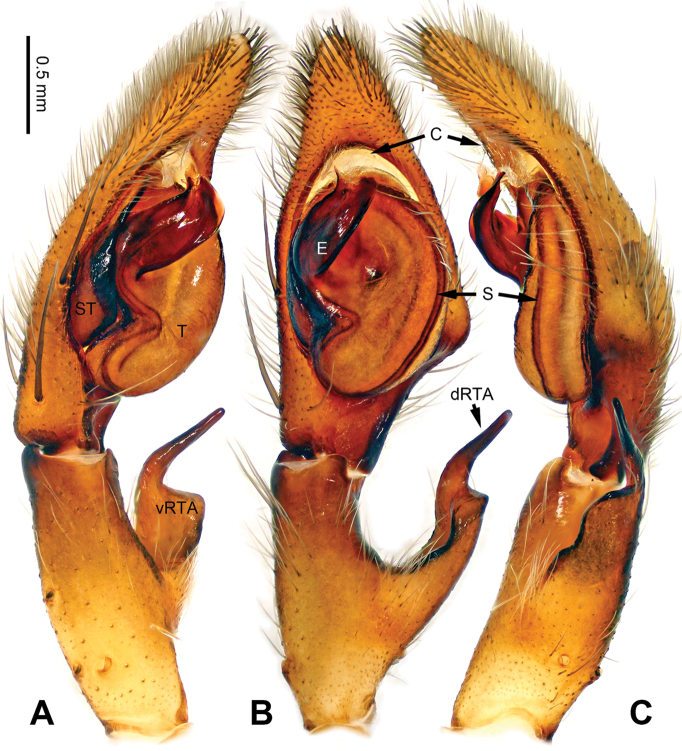
*Pseudopodazixiensis* Zhao & Li, sp. n., left palp of male holotype. **A** Prolateral view **B** Ventral view **C** Retrolateral view. Scale bar equal for **A, B, C**.

**Figure 35.**
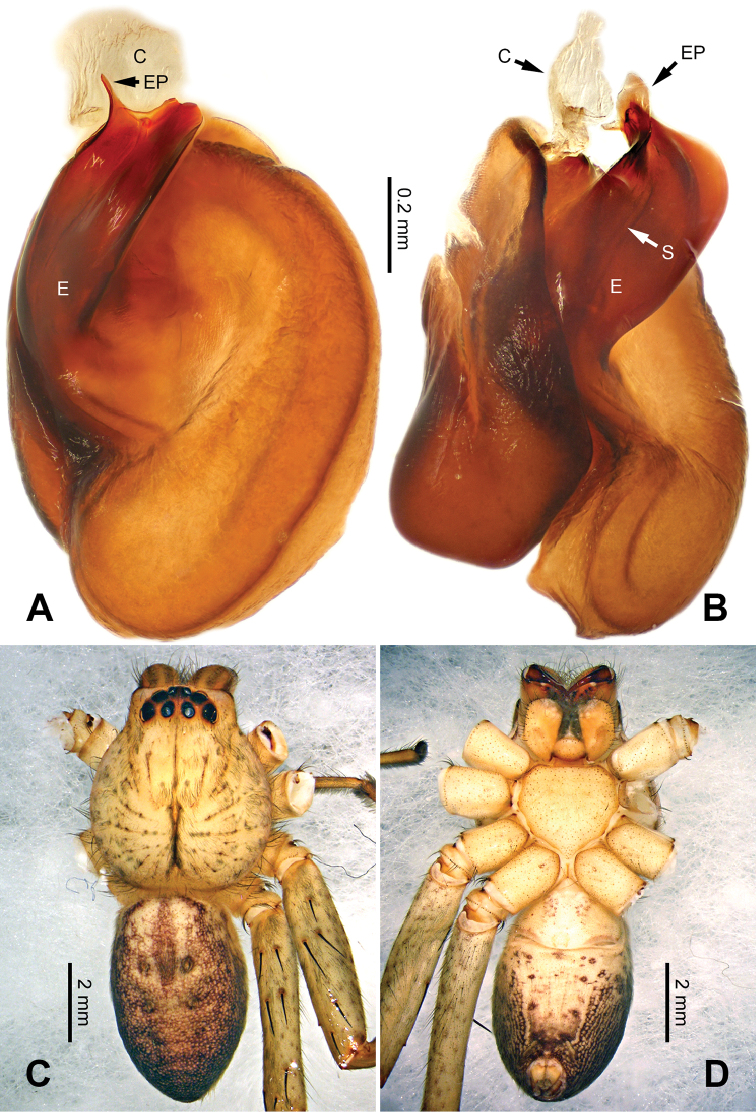
*Pseudopodazixiensis* Zhao & Li, sp. n., male holotype. **A** Bulb, ventral view **B** Bulb, dorsal view **C** Habitus, dorsal view **D** Habitus, ventral view. Scale bar equal for **A, B**.

**Figure 36.**
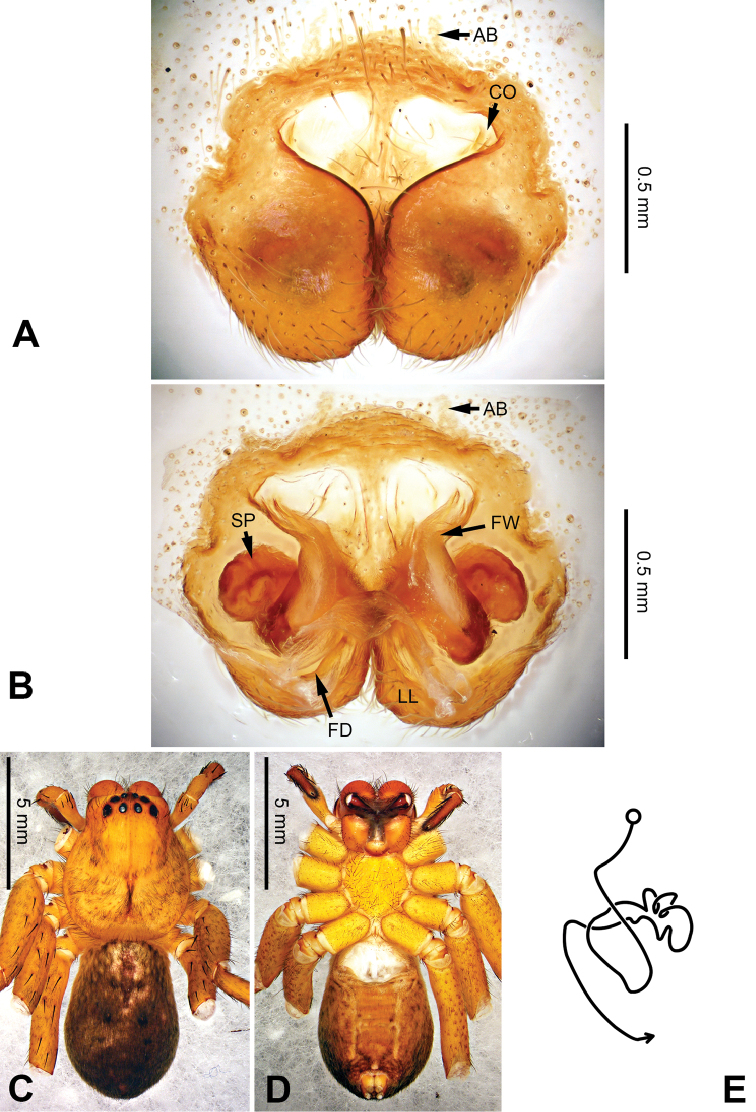
*Pseudopodazixiensis* Zhao & Li, sp. n., paratype female. **A** Epigyne, ventral view **B** Vulva, dorsal view **C** Habitus, dorsal view **D** Habitus, ventral view **E** Schematic course of internal duct system, dorsal view.

**Figure 37.**
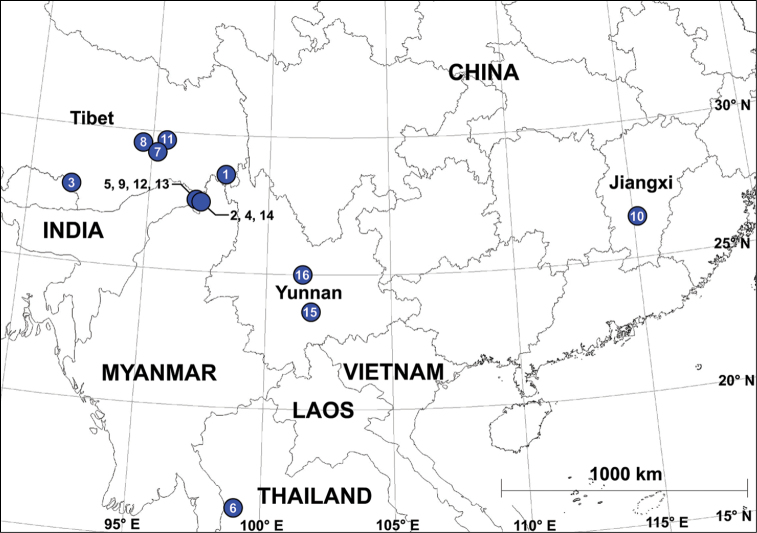
Distribution map of the sixteen new species from the genus *Pseudopoda*. The numbers represent the different species **1***P.chayuensis* Zhao & Li, sp. n. **2***P.colubrina* Zhao & Li, sp. n. **3***P.conaensis* Zhao & Li, sp. n. **4***P.daxing* Zhao & Li, sp. n. **5***P.gexiao* Zhao & Li, sp. n. **6***P.maeklongensis* Zhao & Li, sp. n. **7***P.medogensis* Zhao & Li, sp. n. **8***P.nyingchiensis* Zhao & Li, sp. n. **9***P.putaoensis* Zhao & Li, sp. n. **10***P.shacunensis* Zhao & Li, sp. n. **11***P.shuo* Zhao & Li, sp. n. **12***P.subbirmanica* Zhao & Li, sp. n. **13***P.titan* Zhao & Li, sp. n. **14***P.xia* Zhao & Li, sp. n. **15***P.yuanjiangensis* Zhao & Li, sp. n. **16***P.zixiensis* Zhao & Li, sp. n.

Figs 34 , 35 , 36 , 37

###### Type material.

**Holotype** ♂: China, Yunnan Province, Chuxiong City, Zixi Mountain, 25°00.602'N, 101°24.386'E, 2445 m, VI 2017, Z. Chen. **Paratype**: 1♀, same data as holotype.

###### Etymology.

The specific name refers to the type locality; adjective.

###### Diagnosis.

Median-sized *Pseudopoda* species. Male resembles *P.sinapophysis* Jäger & Vedel, 2007 (see Jäger and Vedel 2007: 3, figs 1–6) and *P.mediana* Quan, Zhong & Liu, 2014 (see Quan et al. 2014: 562, figs 6A–C, 7A–C, 8A–D, 9A–C) by: embolus is curved, with its tip pointing back dorsally (Figure 35B). It can be distinguished from the two congeners by the following combination of characters: 1. cymbium short and blunt (Figure 34B; elongated and slender in *P.sinapophysis* and *P.mediana*); 2. prolateral rim of embolus extended and forming an embolic projection near the tip (Figure 35A, B); 3. dRTA finger-like (Figure 34A–C; broadened in *P.mediana*).

Female resembles *P.cangschana* Jäger & Vedel, 2007 (see Jäger and Vedel 2007: 19, figs 66–72), *P.gongschana* Jäger & Vedel, 2007 (see Jäger and Vedel 2007: 6, figs 10–15) and *P.albolineata* Jäger, 2001 (see Jäger 2001: 83, fig. 46a–o) in ventral view by the similar shape of lateral lobes, but can be distinguished from the three congeners by the following combination of characters: 1. lateral loops of internal duct system (spermathecae) distinct, visible in dorsal view (Figure 36B; spermatheca hidden behind first winding in *P.gongschana*); 2. first winding strongly bent (Figure 36B, E; almost straight in *P.cangschana* and *P.albolineata*).

###### Description.

Male (holotype). Body length 10.5, DS length 5.0, DS width 4.4, OS length 5.5, OS width 3.2. Eyes: AME 0.17, ALE 0.29, PME 0.22, PLE 0.32, AME-AME 0.17, AME-ALE 0.08, PME-PME 0.26, PME-PLE 0.40, AME-PME 0.37, ALE-PLE 0.35, CH AME 0.38, CH ALE 0.30. Spination: palp 131, 101, 2111; legs: femur III 323, IV 331; patella III-IV 101; tibia III-IV 2026; metatarsus III 3025, IV 3037. Measurements of palp and legs: palp 7.3 (2.5, 1.1, 1.4, -, 2.3), leg I -, leg II -, leg III 19.6 (5.4, 2.2, 5.3, 5.1, 1.6), leg IV 21.9 (6.0, 2.1, 5.5, 6.5, 1.8). Promargin of chelicerae with three teeth, retromargin with four teeth. Cheliceral furrow with ca. 25 denticles.

Palp as in diagnosis. Cymbium sub-triangular, with distinct retrolateral bulge. RTA arising basally to mesially from tibia, vRTA humble and broad (Figure 34A–C). Sperm duct running submarginally retrolaterally in tegulum. Embolus broad and sickle-shaped, arising from tegulum at 10 o’clock position. Conductor arising from tegulum at 12 o’clock position, leaning slightly prolaterally (Figure 35A, B).

Coloration in ethanol: carapace yellowish brown. Radial furrows and fovea dark brown. Dorsal opisthosoma reddish brown. Ventral opisthosoma with a pair of bright longitudinal lines. Legs yellowish brown, with randomly distributed reddish brown dots (Figure 35C, D).

Female (paratype). Body length 11.5, DS length 5.5, DS width 4.7, OS length 6.0, OS width 4.2. Eyes: AME 0.21, ALE 0.32, PME 0.24, PLE 0.32, AME-AME 0.25, AME-ALE 0.13, PME-PME 0.33, PME-PLE 0.50, AME-PME 0.43, ALE-PLE 0.42, CH AME 0.50, CH ALE 0.33. Spination: palp 131, 101, 2121, 1014; legs: femur II 323, III 322, IV 331; patella II-IV 001; tibia II-III 2026, IV 2025; metatarsus I-II 1014, III 3015, IV 3037. Measurements of palp and legs: palp 7.2 (2.2, 1.2, 1.6, -, 2.2), leg I - (-, -, -, 4.0, 1.6), leg II 18.5 (5.3, 2.7, 4.6, 4.3, 1.6), leg III 15.3 (4.5, 2.1,4.0, 3.4, 1.3), leg IV 17.4 (5.0, 2.0, 4.3, 4.5, 1.6). Promargin of chelicerae with three teeth, retromargin with four teeth. Cheliceral furrow with ca. 30 denticles.

Epigyne as in diagnosis. Epigynal field with nearly equal length in transverse and longitudinal axis. Anterior bands distinct, anterior margin slightly trilobate. Lateral lobes longer in longitudinal axis. Lateral lobes converged on the central axis, with both anterior and posterior part V-shaped. Spermathecae exposed in dorsal view. Spermathecae oval, with coiling ducts embedded (Figure 36B, E).

Coloration in ethanol: as in male, but generally darker (Figure 36C, D).

###### Distribution.

Known only from the type locality.

## Supplementary Material

XML Treatment for Pseudopoda

XML Treatment for Pseudopoda chayuensis

XML Treatment for Pseudopoda colubrina

XML Treatment for Pseudopoda conaensis

XML Treatment for Pseudopoda daxing

XML Treatment for Pseudopoda gexiao

XML Treatment for Pseudopoda maeklongensis

XML Treatment for Pseudopoda medogensis

XML Treatment for Pseudopoda nyingchiensis

XML Treatment for Pseudopoda putaoensis

XML Treatment for Pseudopoda shacunensis

XML Treatment for Pseudopoda shuo

XML Treatment for Pseudopoda subbirmanica

XML Treatment for Pseudopoda titan

XML Treatment for Pseudopoda xia

XML Treatment for Pseudopoda yuanjiangensis

XML Treatment for Pseudopoda zixiensis
